# Reconstruction of primary vertices at the ATLAS experiment in Run 1 proton–proton collisions at the LHC

**DOI:** 10.1140/epjc/s10052-017-4887-5

**Published:** 2017-05-19

**Authors:** M. Aaboud, G. Aad, B. Abbott, J. Abdallah, O. Abdinov, B. Abeloos, R. Aben, O. S. AbouZeid, N. L. Abraham, H. Abramowicz, H. Abreu, R. Abreu, Y. Abulaiti, B. S. Acharya, L. Adamczyk, D. L. Adams, J. Adelman, S. Adomeit, T. Adye, A. A. Affolder, T. Agatonovic-Jovin, J. Agricola, J. A. Aguilar-Saavedra, S. P. Ahlen, F. Ahmadov, G. Aielli, H. Akerstedt, T. P. A. Åkesson, A. V. Akimov, G. L. Alberghi, J. Albert, S. Albrand, M. J. Alconada Verzini, M. Aleksa, I. N. Aleksandrov, C. Alexa, G. Alexander, T. Alexopoulos, M. Alhroob, B. Ali, M. Aliev, G. Alimonti, J. Alison, S. P. Alkire, B. M. M. Allbrooke, B. W. Allen, P. P. Allport, A. Aloisio, A. Alonso, F. Alonso, C. Alpigiani, M. Alstaty, B. Alvarez Gonzalez, D. Álvarez Piqueras, M. G. Alviggi, B. T. Amadio, K. Amako, Y. Amaral Coutinho, C. Amelung, D. Amidei, S. P. Amor Dos Santos, A. Amorim, S. Amoroso, G. Amundsen, C. Anastopoulos, L. S. Ancu, N. Andari, T. Andeen, C. F. Anders, G. Anders, J. K. Anders, K. J. Anderson, A. Andreazza, V. Andrei, S. Angelidakis, I. Angelozzi, P. Anger, A. Angerami, F. Anghinolfi, A. V. Anisenkov, N. Anjos, A. Annovi, C. Antel, M. Antonelli, A. Antonov, F. Anulli, M. Aoki, L. Aperio Bella, G. Arabidze, Y. Arai, J. P. Araque, A. T. H. Arce, F. A. Arduh, J.-F. Arguin, S. Argyropoulos, M. Arik, A. J. Armbruster, L. J. Armitage, O. Arnaez, H. Arnold, M. Arratia, O. Arslan, A. Artamonov, G. Artoni, S. Artz, S. Asai, N. Asbah, A. Ashkenazi, B. Åsman, L. Asquith, K. Assamagan, R. Astalos, M. Atkinson, N. B. Atlay, K. Augsten, G. Avolio, B. Axen, M. K. Ayoub, G. Azuelos, M. A. Baak, A. E. Baas, M. J. Baca, H. Bachacou, K. Bachas, M. Backes, M. Backhaus, P. Bagiacchi, P. Bagnaia, Y. Bai, J. T. Baines, O. K. Baker, E. M. Baldin, P. Balek, T. Balestri, F. Balli, W. K. Balunas, E. Banas, Sw. Banerjee, A. A. E. Bannoura, L. Barak, E. L. Barberio, D. Barberis, M. Barbero, T. Barillari, M.-S. Barisits, T. Barklow, N. Barlow, S. L. Barnes, B. M. Barnett, R. M. Barnett, Z. Barnovska-Blenessy, A. Baroncelli, G. Barone, A. J. Barr, L. Barranco Navarro, F. Barreiro, J. Barreiro Guimarães da Costa, R. Bartoldus, A. E. Barton, P. Bartos, A. Basalaev, A. Bassalat, R. L. Bates, S. J. Batista, J. R. Batley, M. Battaglia, M. Bauce, F. Bauer, H. S. Bawa, J. B. Beacham, M. D. Beattie, T. Beau, P. H. Beauchemin, P. Bechtle, H. P. Beck, K. Becker, M. Becker, M. Beckingham, C. Becot, A. J. Beddall, A. Beddall, V. A. Bednyakov, M. Bedognetti, C. P. Bee, L. J. Beemster, T. A. Beermann, M. Begel, J. K. Behr, C. Belanger-Champagne, A. S. Bell, G. Bella, L. Bellagamba, A. Bellerive, M. Bellomo, K. Belotskiy, O. Beltramello, N. L. Belyaev, O. Benary, D. Benchekroun, M. Bender, K. Bendtz, N. Benekos, Y. Benhammou, E. Benhar Noccioli, J. Benitez, D. P. Benjamin, J. R. Bensinger, S. Bentvelsen, L. Beresford, M. Beretta, D. Berge, E. Bergeaas Kuutmann, N. Berger, J. Beringer, S. Berlendis, N. R. Bernard, C. Bernius, F. U. Bernlochner, T. Berry, P. Berta, C. Bertella, G. Bertoli, F. Bertolucci, I. A. Bertram, C. Bertsche, D. Bertsche, G. J. Besjes, O. Bessidskaia Bylund, M. Bessner, N. Besson, C. Betancourt, S. Bethke, A. J. Bevan, W. Bhimji, R. M. Bianchi, L. Bianchini, M. Bianco, O. Biebel, D. Biedermann, R. Bielski, N. V. Biesuz, M. Biglietti, J. Bilbao De Mendizabal, H. Bilokon, M. Bindi, S. Binet, A. Bingul, C. Bini, S. Biondi, D. M. Bjergaard, C. W. Black, J. E. Black, K. M. Black, D. Blackburn, R. E. Blair, J.-B. Blanchard, J. E. Blanco, T. Blazek, I. Bloch, C. Blocker, W. Blum, U. Blumenschein, S. Blunier, G. J. Bobbink, V. S. Bobrovnikov, S. S. Bocchetta, A. Bocci, C. Bock, M. Boehler, D. Boerner, J. A. Bogaerts, D. Bogavac, A. G. Bogdanchikov, C. Bohm, V. Boisvert, P. Bokan, T. Bold, A. S. Boldyrev, M. Bomben, M. Bona, M. Boonekamp, A. Borisov, G. Borissov, J. Bortfeldt, D. Bortoletto, V. Bortolotto, D. Boscherini, M. Bosman, J. D. Bossio Sola, J. Boudreau, J. Bouffard, E. V. Bouhova-Thacker, D. Boumediene, C. Bourdarios, S. K. Boutle, A. Boveia, J. Boyd, I. R. Boyko, J. Bracinik, A. Brandt, G. Brandt, O. Brandt, U. Bratzler, B. Brau, J. E. Brau, H. M. Braun, W. D. Breaden Madden, K. Brendlinger, A. J. Brennan, L. Brenner, R. Brenner, S. Bressler, T. M. Bristow, D. Britton, D. Britzger, F. M. Brochu, I. Brock, R. Brock, G. Brooijmans, T. Brooks, W. K. Brooks, J. Brosamer, E. Brost, J. H Broughton, P. A. Bruckman de Renstrom, D. Bruncko, R. Bruneliere, A. Bruni, G. Bruni, L. S. Bruni, BH Brunt, M. Bruschi, N. Bruscino, P. Bryant, L. Bryngemark, T. Buanes, Q. Buat, P. Buchholz, A. G. Buckley, I. A. Budagov, F. Buehrer, M. K. Bugge, O. Bulekov, D. Bullock, H. Burckhart, S. Burdin, C. D. Burgard, B. Burghgrave, K. Burka, S. Burke, I. Burmeister, J. T. P. Burr, E. Busato, D. Büscher, V. Büscher, P. Bussey, J. M. Butler, C. M. Buttar, J. M. Butterworth, P. Butti, W. Buttinger, A. Buzatu, A. R. Buzykaev, S. Cabrera Urbán, D. Caforio, V. M. Cairo, O. Cakir, N. Calace, P. Calafiura, A. Calandri, G. Calderini, P. Calfayan, G. Callea, L. P. Caloba, S. Calvente Lopez, D. Calvet, S. Calvet, T. P. Calvet, R. Camacho Toro, S. Camarda, P. Camarri, D. Cameron, R. Caminal Armadans, C. Camincher, S. Campana, M. Campanelli, A. Camplani, A. Campoverde, V. Canale, A. Canepa, M. Cano Bret, J. Cantero, R. Cantrill, T. Cao, M. D. M. Capeans Garrido, I. Caprini, M. Caprini, M. Capua, R. Caputo, R. M. Carbone, R. Cardarelli, F. Cardillo, I. Carli, T. Carli, G. Carlino, L. Carminati, S. Caron, E. Carquin, G. D. Carrillo-Montoya, J. R. Carter, J. Carvalho, D. Casadei, M. P. Casado, M. Casolino, D. W. Casper, E. Castaneda-Miranda, R. Castelijn, A. Castelli, V. Castillo Gimenez, N. F. Castro, A. Catinaccio, J. R. Catmore, A. Cattai, J. Caudron, V. Cavaliere, E. Cavallaro, D. Cavalli, M. Cavalli-Sforza, V. Cavasinni, F. Ceradini, L. Cerda Alberich, B. C. Cerio, A. S. Cerqueira, A. Cerri, L. Cerrito, F. Cerutti, M. Cerv, A. Cervelli, S. A. Cetin, A. Chafaq, D. Chakraborty, S. K. Chan, Y. L. Chan, P. Chang, J. D. Chapman, D. G. Charlton, A. Chatterjee, C. C. Chau, C. A. Chavez Barajas, S. Che, S. Cheatham, A. Chegwidden, S. Chekanov, S. V. Chekulaev, G. A. Chelkov, M. A. Chelstowska, C. Chen, H. Chen, K. Chen, S. Chen, S. Chen, X. Chen, Y. Chen, H. C. Cheng, H. J. Cheng, Y. Cheng, A. Cheplakov, E. Cheremushkina, R. Cherkaoui El Moursli, V. Chernyatin, E. Cheu, L. Chevalier, V. Chiarella, G. Chiarelli, G. Chiodini, A. S. Chisholm, A. Chitan, M. V. Chizhov, K. Choi, A. R. Chomont, S. Chouridou, B. K. B. Chow, V. Christodoulou, D. Chromek-Burckhart, J. Chudoba, A. J. Chuinard, J. J. Chwastowski, L. Chytka, G. Ciapetti, A. K. Ciftci, D. Cinca, V. Cindro, I. A. Cioara, C. Ciocca, A. Ciocio, F. Cirotto, Z. H. Citron, M. Citterio, M. Ciubancan, A. Clark, B. L. Clark, M. R. Clark, P. J. Clark, R. N. Clarke, C. Clement, Y. Coadou, M. Cobal, A. Coccaro, J. Cochran, L. Coffey, L. Colasurdo, B. Cole, A. P. Colijn, J. Collot, T. Colombo, G. Compostella, P. Conde Muiño, E. Coniavitis, S. H. Connell, I. A. Connelly, V. Consorti, S. Constantinescu, G. Conti, F. Conventi, M. Cooke, B. D. Cooper, A. M. Cooper-Sarkar, K. J. R. Cormier, T. Cornelissen, M. Corradi, F. Corriveau, A. Corso-Radu, A. Cortes-Gonzalez, G. Cortiana, G. Costa, M. J. Costa, D. Costanzo, G. Cottin, G. Cowan, B. E. Cox, K. Cranmer, S. J. Crawley, G. Cree, S. Crépé-Renaudin, F. Crescioli, W. A. Cribbs, M. Crispin Ortuzar, M. Cristinziani, V. Croft, G. Crosetti, T. Cuhadar Donszelmann, J. Cummings, M. Curatolo, J. Cúth, C. Cuthbert, H. Czirr, P. Czodrowski, G. D’amen, S. D’Auria, M. D’Onofrio, M. J. Da Cunha Sargedas De Sousa, C. Da Via, W. Dabrowski, T. Dado, T. Dai, O. Dale, F. Dallaire, C. Dallapiccola, M. Dam, J. R. Dandoy, N. P. Dang, A. C. Daniells, N. S. Dann, M. Danninger, M. Dano Hoffmann, V. Dao, G. Darbo, S. Darmora, J. Dassoulas, A. Dattagupta, W. Davey, C. David, T. Davidek, M. Davies, P. Davison, E. Dawe, I. Dawson, R. K. Daya-Ishmukhametova, K. De, R. de Asmundis, A. De Benedetti, S. De Castro, S. De Cecco, N. De Groot, P. de Jong, H. De la Torre, F. De Lorenzi, A. De Maria, D. De Pedis, A. De Salvo, U. De Sanctis, A. De Santo, J. B. De Vivie De Regie, W. J. Dearnaley, R. Debbe, C. Debenedetti, D. V. Dedovich, N. Dehghanian, I. Deigaard, M. Del Gaudio, J. Del Peso, T. Del Prete, D. Delgove, F. Deliot, C. M. Delitzsch, M. Deliyergiyev, A. Dell’Acqua, L. Dell’Asta, M. Dell’Orso, M. Della Pietra, D. della Volpe, M. Delmastro, P. A. Delsart, D. A. DeMarco, S. Demers, M. Demichev, A. Demilly, S. P. Denisov, D. Denysiuk, D. Derendarz, J. E. Derkaoui, F. Derue, P. Dervan, K. Desch, C. Deterre, K. Dette, P. O. Deviveiros, A. Dewhurst, S. Dhaliwal, A. Di Ciaccio, L. Di Ciaccio, W. K. Di Clemente, C. Di Donato, A. Di Girolamo, B. Di Girolamo, B. Di Micco, R. Di Nardo, A. Di Simone, R. Di Sipio, D. Di Valentino, C. Diaconu, M. Diamond, F. A. Dias, M. A. Diaz, E. B. Diehl, J. Dietrich, S. Diglio, A. Dimitrievska, J. Dingfelder, P. Dita, S. Dita, F. Dittus, F. Djama, T. Djobava, J. I. Djuvsland, M. A. B. do Vale, D. Dobos, M. Dobre, C. Doglioni, T. Dohmae, J. Dolejsi, Z. Dolezal, B. A. Dolgoshein, M. Donadelli, S. Donati, P. Dondero, J. Donini, J. Dopke, A. Doria, M. T. Dova, A. T. Doyle, E. Drechsler, M. Dris, Y. Du, J. Duarte-Campderros, E. Duchovni, G. Duckeck, O. A. Ducu, D. Duda, A. Dudarev, E. M. Duffield, L. Duflot, L. Duguid, M. Dührssen, M. Dumancic, M. Dunford, H. Duran Yildiz, M. Düren, A. Durglishvili, D. Duschinger, B. Dutta, M. Dyndal, C. Eckardt, K. M. Ecker, R. C. Edgar, N. C. Edwards, T. Eifert, G. Eigen, K. Einsweiler, T. Ekelof, M. El Kacimi, V. Ellajosyula, M. Ellert, S. Elles, F. Ellinghaus, A. A. Elliot, N. Ellis, J. Elmsheuser, M. Elsing, D. Emeliyanov, Y. Enari, O. C. Endner, M. Endo, J. S. Ennis, J. Erdmann, A. Ereditato, G. Ernis, J. Ernst, M. Ernst, S. Errede, E. Ertel, M. Escalier, H. Esch, C. Escobar, B. Esposito, A. I. Etienvre, E. Etzion, H. Evans, A. Ezhilov, F. Fabbri, L. Fabbri, G. Facini, R. M. Fakhrutdinov, S. Falciano, R. J. Falla, J. Faltova, Y. Fang, M. Fanti, A. Farbin, A. Farilla, C. Farina, E. M. Farina, T. Farooque, S. Farrell, S. M. Farrington, P. Farthouat, F. Fassi, P. Fassnacht, D. Fassouliotis, M. Faucci Giannelli, A. Favareto, W. J. Fawcett, L. Fayard, O. L. Fedin, W. Fedorko, S. Feigl, L. Feligioni, C. Feng, E. J. Feng, H. Feng, A. B. Fenyuk, L. Feremenga, P. Fernandez Martinez, S. Fernandez Perez, J. Ferrando, A. Ferrari, P. Ferrari, R. Ferrari, D. E. Ferreira de Lima, A. Ferrer, D. Ferrere, C. Ferretti, A. Ferretto Parodi, F. Fiedler, A. Filipčič, M. Filipuzzi, F. Filthaut, M. Fincke-Keeler, K. D. Finelli, M. C. N. Fiolhais, L. Fiorini, A. Firan, A. Fischer, C. Fischer, J. Fischer, W. C. Fisher, N. Flaschel, I. Fleck, P. Fleischmann, G. T. Fletcher, R. R. M. Fletcher, T. Flick, A. Floderus, L. R. Flores Castillo, M. J. Flowerdew, G. T. Forcolin, A. Formica, A. Forti, A. G. Foster, D. Fournier, H. Fox, S. Fracchia, P. Francavilla, M. Franchini, D. Francis, L. Franconi, M. Franklin, M. Frate, M. Fraternali, D. Freeborn, S. M. Fressard-Batraneanu, F. Friedrich, D. Froidevaux, J. A. Frost, C. Fukunaga, T. Fusayasu, J. Fuster, C. Gabaldon, O. Gabizon, A. Gabrielli, A. Gabrielli, G. P. Gach, S. Gadatsch, S. Gadomski, G. Gagliardi, L. G. Gagnon, P. Gagnon, C. Galea, B. Galhardo, E. J. Gallas, B. J. Gallop, P. Gallus, G. Galster, K. K. Gan, J. Gao, Y. Gao, Y. S. Gao, F. M. Garay Walls, C. García, J. E. García Navarro, M. Garcia-Sciveres, R. W. Gardner, N. Garelli, V. Garonne, A. Gascon Bravo, C. Gatti, A. Gaudiello, G. Gaudio, B. Gaur, L. Gauthier, I. L. Gavrilenko, C. Gay, G. Gaycken, E. N. Gazis, Z. Gecse, C. N. P. Gee, Ch. Geich-Gimbel, M. Geisen, M. P. Geisler, C. Gemme, M. H. Genest, C. Geng, S. Gentile, C. Gentsos, S. George, D. Gerbaudo, A. Gershon, S. Ghasemi, H. Ghazlane, M. Ghneimat, B. Giacobbe, S. Giagu, P. Giannetti, B. Gibbard, S. M. Gibson, M. Gignac, M. Gilchriese, T. P. S. Gillam, D. Gillberg, G. Gilles, D. M. Gingrich, N. Giokaris, M. P. Giordani, F. M. Giorgi, F. M. Giorgi, P. F. Giraud, P. Giromini, D. Giugni, F. Giuli, C. Giuliani, M. Giulini, B. K. Gjelsten, S. Gkaitatzis, I. Gkialas, E. L. Gkougkousis, L. K. Gladilin, C. Glasman, J. Glatzer, P. C. F. Glaysher, A. Glazov, M. Goblirsch-Kolb, J. Godlewski, S. Goldfarb, T. Golling, D. Golubkov, A. Gomes, R. Gonçalo, J. Goncalves Pinto Firmino Da Costa, G. Gonella, L. Gonella, A. Gongadze, S. González de la Hoz, G. Gonzalez Parra, S. Gonzalez-Sevilla, L. Goossens, P. A. Gorbounov, H. A. Gordon, I. Gorelov, B. Gorini, E. Gorini, A. Gorišek, E. Gornicki, A. T. Goshaw, C. Gössling, M. I. Gostkin, C. R. Goudet, D. Goujdami, A. G. Goussiou, N. Govender, E. Gozani, L. Graber, I. Grabowska-Bold, P. O. J. Gradin, P. Grafström, J. Gramling, E. Gramstad, S. Grancagnolo, V. Gratchev, P. M. Gravila, H. M. Gray, E. Graziani, Z. D. Greenwood, C. Grefe, K. Gregersen, I. M. Gregor, P. Grenier, K. Grevtsov, J. Griffiths, A. A. Grillo, K. Grimm, S. Grinstein, Ph. Gris, J.-F. Grivaz, S. Groh, J. P. Grohs, E. Gross, J. Grosse-Knetter, G. C. Grossi, Z. J. Grout, L. Guan, W. Guan, J. Guenther, F. Guescini, D. Guest, O. Gueta, E. Guido, T. Guillemin, S. Guindon, U. Gul, C. Gumpert, J. Guo, Y. Guo, R. Gupta, S. Gupta, G. Gustavino, P. Gutierrez, N. G. Gutierrez Ortiz, C. Gutschow, C. Guyot, C. Gwenlan, C. B. Gwilliam, A. Haas, C. Haber, H. K. Hadavand, A. Hadef, P. Haefner, S. Hageböck, Z. Hajduk, H. Hakobyan, M. Haleem, J. Haley, G. Halladjian, G. D. Hallewell, K. Hamacher, P. Hamal, K. Hamano, A. Hamilton, G. N. Hamity, P. G. Hamnett, L. Han, K. Hanagaki, K. Hanawa, M. Hance, B. Haney, P. Hanke, R. Hanna, J. B. Hansen, J. D. Hansen, M. C. Hansen, P. H. Hansen, K. Hara, A. S. Hard, T. Harenberg, F. Hariri, S. Harkusha, R. D. Harrington, P. F. Harrison, N. M. Hartmann, M. Hasegawa, Y. Hasegawa, A. Hasib, S. Hassani, S. Haug, R. Hauser, L. Hauswald, M. Havranek, C. M. Hawkes, R. J. Hawkings, D. Hayden, C. P. Hays, J. M. Hays, H. S. Hayward, S. J. Haywood, S. J. Head, T. Heck, V. Hedberg, L. Heelan, K. K. Heidegger, S. Heim, T. Heim, B. Heinemann, J. J. Heinrich, L. Heinrich, C. Heinz, J. Hejbal, L. Helary, S. Hellman, C. Helsens, J. Henderson, R. C. W. Henderson, Y. Heng, S. Henkelmann, A. M. Henriques Correia, S. Henrot-Versille, G. H. Herbert, Y. Hernández Jiménez, G. Herten, R. Hertenberger, L. Hervas, G. G. Hesketh, N. P. Hessey, J. W. Hetherly, R. Hickling, E. Higón-Rodriguez, E. Hill, J. C. Hill, K. H. Hiller, S. J. Hillier, I. Hinchliffe, E. Hines, R. R. Hinman, M. Hirose, D. Hirschbuehl, J. Hobbs, N. Hod, M. C. Hodgkinson, P. Hodgson, A. Hoecker, M. R. Hoeferkamp, F. Hoenig, D. Hohn, T. R. Holmes, M. Homann, T. M. Hong, B. H. Hooberman, W. H. Hopkins, Y. Horii, A. J. Horton, J.-Y. Hostachy, S. Hou, A. Hoummada, J. Howarth, M. Hrabovsky, I. Hristova, J. Hrivnac, T. Hryn’ova, A. Hrynevich, C. Hsu, P. J. Hsu, S.-C. Hsu, D. Hu, Q. Hu, Y. Huang, Z. Hubacek, F. Hubaut, F. Huegging, T. B. Huffman, E. W. Hughes, G. Hughes, M. Huhtinen, P. Huo, N. Huseynov, J. Huston, J. Huth, G. Iacobucci, G. Iakovidis, I. Ibragimov, L. Iconomidou-Fayard, E. Ideal, P. Iengo, O. Igonkina, T. Iizawa, Y. Ikegami, M. Ikeno, Y. Ilchenko, D. Iliadis, N. Ilic, T. Ince, G. Introzzi, P. Ioannou, M. Iodice, K. Iordanidou, V. Ippolito, N. Ishijima, M. Ishino, M. Ishitsuka, R. Ishmukhametov, C. Issever, S. Istin, F. Ito, J. M. Iturbe Ponce, R. Iuppa, W. Iwanski, H. Iwasaki, J. M. Izen, V. Izzo, S. Jabbar, B. Jackson, M. Jackson, P. Jackson, V. Jain, K. B. Jakobi, K. Jakobs, S. Jakobsen, T. Jakoubek, D. O. Jamin, D. K. Jana, E. Jansen, R. Jansky, J. Janssen, M. Janus, G. Jarlskog, N. Javadov, T. Javůrek, M. Javurkova, F. Jeanneau, L. Jeanty, G.-Y. Jeng, D. Jennens, P. Jenni, J. Jentzsch, C. Jeske, S. Jézéquel, H. Ji, J. Jia, H. Jiang, Y. Jiang, S. Jiggins, J. Jimenez Pena, S. Jin, A. Jinaru, O. Jinnouchi, P. Johansson, K. A. Johns, W. J. Johnson, K. Jon-And, G. Jones, R. W. L. Jones, S. Jones, T. J. Jones, J. Jongmanns, P. M. Jorge, J. Jovicevic, X. Ju, A. Juste Rozas, M. K. Köhler, A. Kaczmarska, M. Kado, H. Kagan, M. Kagan, S. J. Kahn, E. Kajomovitz, C. W. Kalderon, A. Kaluza, S. Kama, A. Kamenshchikov, N. Kanaya, S. Kaneti, L. Kanjir, V. A. Kantserov, J. Kanzaki, B. Kaplan, L. S. Kaplan, A. Kapliy, D. Kar, K. Karakostas, A. Karamaoun, N. Karastathis, M. J. Kareem, E. Karentzos, M. Karnevskiy, S. N. Karpov, Z. M. Karpova, K. Karthik, V. Kartvelishvili, A. N. Karyukhin, K. Kasahara, L. Kashif, R. D. Kass, A. Kastanas, Y. Kataoka, C. Kato, A. Katre, J. Katzy, K. Kawade, K. Kawagoe, T. Kawamoto, G. Kawamura, S. Kazama, V. F. Kazanin, R. Keeler, R. Kehoe, J. S. Keller, J. J. Kempster, H. Keoshkerian, O. Kepka, B. P. Kerševan, S. Kersten, R. A. Keyes, M. Khader, F. Khalil-zada, A. Khanov, A. G. Kharlamov, T. J. Khoo, V. Khovanskiy, E. Khramov, J. Khubua, S. Kido, H. Y. Kim, S. H. Kim, Y. K. Kim, N. Kimura, O. M. Kind, B. T. King, M. King, S. B. King, J. Kirk, A. E. Kiryunin, T. Kishimoto, D. Kisielewska, F. Kiss, K. Kiuchi, O. Kivernyk, E. Kladiva, M. H. Klein, M. Klein, U. Klein, K. Kleinknecht, P. Klimek, A. Klimentov, R. Klingenberg, J. A. Klinger, T. Klioutchnikova, E.-E. Kluge, P. Kluit, S. Kluth, J. Knapik, E. Kneringer, E. B. F. G. Knoops, A. Knue, A. Kobayashi, D. Kobayashi, T. Kobayashi, M. Kobel, M. Kocian, P. Kodys, T. Koffas, E. Koffeman, T. Koi, H. Kolanoski, M. Kolb, I. Koletsou, A. A. Komar, Y. Komori, T. Kondo, N. Kondrashova, K. Köneke, A. C. König, T. Kono, R. Konoplich, N. Konstantinidis, R. Kopeliansky, S. Koperny, L. Köpke, A. K. Kopp, K. Korcyl, K. Kordas, A. Korn, A. A. Korol, I. Korolkov, E. V. Korolkova, O. Kortner, S. Kortner, T. Kosek, V. V. Kostyukhin, A. Kotwal, A. Kourkoumeli-Charalampidi, C. Kourkoumelis, V. Kouskoura, A. B. Kowalewska, R. Kowalewski, T. Z. Kowalski, C. Kozakai, W. Kozanecki, A. S. Kozhin, V. A. Kramarenko, G. Kramberger, D. Krasnopevtsev, M. W. Krasny, A. Krasznahorkay, J. K. Kraus, A. Kravchenko, M. Kretz, J. Kretzschmar, K. Kreutzfeldt, P. Krieger, K. Krizka, K. Kroeninger, H. Kroha, J. Kroll, J. Kroseberg, J. Krstic, U. Kruchonak, H. Krüger, N. Krumnack, A. Kruse, M. C. Kruse, M. Kruskal, T. Kubota, H. Kucuk, S. Kuday, J. T. Kuechler, S. Kuehn, A. Kugel, F. Kuger, A. Kuhl, T. Kuhl, V. Kukhtin, R. Kukla, Y. Kulchitsky, S. Kuleshov, M. Kuna, T. Kunigo, A. Kupco, H. Kurashige, Y. A. Kurochkin, V. Kus, E. S. Kuwertz, M. Kuze, J. Kvita, T. Kwan, D. Kyriazopoulos, A. La Rosa, J. L. La Rosa Navarro, L. La Rotonda, C. Lacasta, F. Lacava, J. Lacey, H. Lacker, D. Lacour, V. R. Lacuesta, E. Ladygin, R. Lafaye, B. Laforge, T. Lagouri, S. Lai, S. Lammers, W. Lampl, E. Lançon, U. Landgraf, M. P. J. Landon, M. C. Lanfermann, V. S. Lang, J. C. Lange, A. J. Lankford, F. Lanni, K. Lantzsch, A. Lanza, S. Laplace, C. Lapoire, J. F. Laporte, T. Lari, F. Lasagni Manghi, M. Lassnig, P. Laurelli, W. Lavrijsen, A. T. Law, P. Laycock, T. Lazovich, M. Lazzaroni, B. Le, O. Le Dortz, E. Le Guirriec, E. P. Le Quilleuc, M. LeBlanc, T. LeCompte, F. Ledroit-Guillon, C. A. Lee, S. C. Lee, L. Lee, G. Lefebvre, M. Lefebvre, F. Legger, C. Leggett, A. Lehan, G. Lehmann Miotto, X. Lei, W. A. Leight, A. G. Leister, M. A. L. Leite, R. Leitner, D. Lellouch, B. Lemmer, K. J. C. Leney, T. Lenz, B. Lenzi, R. Leone, S. Leone, C. Leonidopoulos, S. Leontsinis, G. Lerner, C. Leroy, A. A. J. Lesage, C. G. Lester, M. Levchenko, J. Levêque, D. Levin, L. J. Levinson, M. Levy, D. Lewis, A. M. Leyko, B. Li, H. Li, H. L. Li, L. Li, L. Li, Q. Li, S. Li, X. Li, Y. Li, Z. Liang, B. Liberti, A. Liblong, P. Lichard, K. Lie, J. Liebal, W. Liebig, A. Limosani, S. C. Lin, T. H. Lin, B. E. Lindquist, A. E. Lionti, E. Lipeles, A. Lipniacka, M. Lisovyi, T. M. Liss, A. Lister, A. M. Litke, B. Liu, D. Liu, H. Liu, H. Liu, J. Liu, J. B. Liu, K. Liu, L. Liu, M. Liu, M. Liu, Y. L. Liu, Y. Liu, M. Livan, A. Lleres, J. Llorente Merino, S. L. Lloyd, F. Lo Sterzo, E. M. Lobodzinska, P. Loch, W. S. Lockman, F. K. Loebinger, A. E. Loevschall-Jensen, K. M. Loew, A. Loginov, T. Lohse, K. Lohwasser, M. Lokajicek, B. A. Long, J. D. Long, R. E. Long, L. Longo, K. A. Looper, L. Lopes, D. Lopez Mateos, B. Lopez Paredes, I. Lopez Paz, A. Lopez Solis, J. Lorenz, N. Lorenzo Martinez, M. Losada, P. J. Lösel, X. Lou, A. Lounis, J. Love, P. A. Love, H. Lu, N. Lu, H. J. Lubatti, C. Luci, A. Lucotte, C. Luedtke, F. Luehring, W. Lukas, L. Luminari, O. Lundberg, B. Lund-Jensen, P. M. Luzi, D. Lynn, R. Lysak, E. Lytken, V. Lyubushkin, H. Ma, L. L. Ma, Y. Ma, G. Maccarrone, A. Macchiolo, C. M. Macdonald, B. Maček, J. Machado Miguens, D. Madaffari, R. Madar, H. J. Maddocks, W. F. Mader, A. Madsen, J. Maeda, S. Maeland, T. Maeno, A. S. Maevskiy, E. Magradze, J. Mahlstedt, C. Maiani, C. Maidantchik, A. A. Maier, T. Maier, A. Maio, S. Majewski, Y. Makida, N. Makovec, B. Malaescu, Pa. Malecki, V. P. Maleev, F. Malek, U. Mallik, D. Malon, C. Malone, S. Maltezos, S. Malyukov, J. Mamuzic, G. Mancini, B. Mandelli, L. Mandelli, I. Mandić, J. Maneira, L. Manhaes de Andrade Filho, J. Manjarres Ramos, A. Mann, A. Manousos, B. Mansoulie, J. D. Mansour, R. Mantifel, M. Mantoani, S. Manzoni, L. Mapelli, G. Marceca, L. March, G. Marchiori, M. Marcisovsky, M. Marjanovic, D. E. Marley, F. Marroquim, S. P. Marsden, Z. Marshall, S. Marti-Garcia, B. Martin, T. A. Martin, V. J. Martin, B. Martin dit Latour, M. Martinez, V. I. Martinez Outschoorn, S. Martin-Haugh, V. S. Martoiu, A. C. Martyniuk, M. Marx, A. Marzin, L. Masetti, T. Mashimo, R. Mashinistov, J. Masik, A. L. Maslennikov, I. Massa, L. Massa, P. Mastrandrea, A. Mastroberardino, T. Masubuchi, P. Mättig, J. Mattmann, J. Maurer, S. J. Maxfield, D. A. Maximov, R. Mazini, S. M. Mazza, N. C. Mc Fadden, G. Mc Goldrick, S. P. Mc Kee, A. McCarn, R. L. McCarthy, T. G. McCarthy, L. I. McClymont, E. F. McDonald, J. A. Mcfayden, G. Mchedlidze, S. J. McMahon, R. A. McPherson, M. Medinnis, S. Meehan, S. Mehlhase, A. Mehta, K. Meier, C. Meineck, B. Meirose, D. Melini, B. R. Mellado Garcia, M. Melo, F. Meloni, A. Mengarelli, S. Menke, E. Meoni, S. Mergelmeyer, P. Mermod, L. Merola, C. Meroni, F. S. Merritt, A. Messina, J. Metcalfe, A. S. Mete, C. Meyer, C. Meyer, J.-P. Meyer, J. Meyer, H. Meyer Zu Theenhausen, F. Miano, R. P. Middleton, S. Miglioranzi, L. Mijović, G. Mikenberg, M. Mikestikova, M. Mikuž, M. Milesi, A. Milic, D. W. Miller, C. Mills, A. Milov, D. A. Milstead, A. A. Minaenko, Y. Minami, I. A. Minashvili, A. I. Mincer, B. Mindur, M. Mineev, Y. Ming, L. M. Mir, K. P. Mistry, T. Mitani, J. Mitrevski, V. A. Mitsou, A. Miucci, P. S. Miyagawa, J. U. Mjörnmark, T. Moa, K. Mochizuki, S. Mohapatra, S. Molander, R. Moles-Valls, R. Monden, M. C. Mondragon, K. Mönig, J. Monk, E. Monnier, A. Montalbano, J. Montejo Berlingen, F. Monticelli, S. Monzani, R. W. Moore, N. Morange, D. Moreno, M. Moreno Llácer, P. Morettini, S. Morgenstern, D. Mori, T. Mori, M. Morii, M. Morinaga, V. Morisbak, S. Moritz, A. K. Morley, G. Mornacchi, J. D. Morris, L. Morvaj, M. Mosidze, J. Moss, K. Motohashi, R. Mount, E. Mountricha, S. V. Mouraviev, E. J. W. Moyse, S. Muanza, R. D. Mudd, F. Mueller, J. Mueller, R. S. P. Mueller, T. Mueller, D. Muenstermann, P. Mullen, G. A. Mullier, F. J. Munoz Sanchez, J. A. Murillo Quijada, W. J. Murray, H. Musheghyan, M. Muškinja, A. G. Myagkov, M. Myska, B. P. Nachman, O. Nackenhorst, K. Nagai, R. Nagai, K. Nagano, Y. Nagasaka, K. Nagata, M. Nagel, E. Nagy, A. M. Nairz, Y. Nakahama, K. Nakamura, T. Nakamura, I. Nakano, H. Namasivayam, R. F. Naranjo Garcia, R. Narayan, D. I. Narrias Villar, I. Naryshkin, T. Naumann, G. Navarro, R. Nayyar, H. A. Neal, P. Yu. Nechaeva, T. J. Neep, P. D. Nef, A. Negri, M. Negrini, S. Nektarijevic, C. Nellist, A. Nelson, S. Nemecek, P. Nemethy, A. A. Nepomuceno, M. Nessi, M. S. Neubauer, M. Neumann, R. M. Neves, P. Nevski, P. R. Newman, D. H. Nguyen, T. Nguyen Manh, R. B. Nickerson, R. Nicolaidou, J. Nielsen, A. Nikiforov, V. Nikolaenko, I. Nikolic-Audit, K. Nikolopoulos, J. K. Nilsen, P. Nilsson, Y. Ninomiya, A. Nisati, R. Nisius, T. Nobe, M. Nomachi, I. Nomidis, T. Nooney, S. Norberg, M. Nordberg, N. Norjoharuddeen, O. Novgorodova, S. Nowak, M. Nozaki, L. Nozka, K. Ntekas, E. Nurse, F. Nuti, F. O’grady, D. C. O’Neil, A. A. O’Rourke, V. O’Shea, F. G. Oakham, H. Oberlack, T. Obermann, J. Ocariz, A. Ochi, I. Ochoa, J. P. Ochoa-Ricoux, S. Oda, S. Odaka, H. Ogren, A. Oh, S. H. Oh, C. C. Ohm, H. Ohman, H. Oide, H. Okawa, Y. Okumura, T. Okuyama, A. Olariu, L. F. Oleiro Seabra, S. A. Olivares Pino, D. Oliveira Damazio, A. Olszewski, J. Olszowska, A. Onofre, K. Onogi, P. U. E. Onyisi, M. J. Oreglia, Y. Oren, D. Orestano, N. Orlando, R. S. Orr, B. Osculati, R. Ospanov, G. Otero y Garzon, H. Otono, M. Ouchrif, F. Ould-Saada, A. Ouraou, K. P. Oussoren, Q. Ouyang, M. Owen, R. E. Owen, V. E. Ozcan, N. Ozturk, K. Pachal, A. Pacheco Pages, L. Pacheco Rodriguez, C. Padilla Aranda, S. Pagan Griso, F. Paige, P. Pais, K. Pajchel, G. Palacino, S. Palazzo, S. Palestini, M. Palka, D. Pallin, A. Palma, E. St. Panagiotopoulou, C. E. Pandini, J. G. Panduro Vazquez, P. Pani, S. Panitkin, D. Pantea, L. Paolozzi, Th. D. Papadopoulou, K. Papageorgiou, A. Paramonov, D. Paredes Hernandez, A. J. Parker, M. A. Parker, K. A. Parker, F. Parodi, J. A. Parsons, U. Parzefall, V. R. Pascuzzi, E. Pasqualucci, S. Passaggio, Fr. Pastore, G. Pásztor, S. Pataraia, J. R. Pater, T. Pauly, J. Pearce, B. Pearson, L. E. Pedersen, S. Pedraza Lopez, R. Pedro, S. V. Peleganchuk, D. Pelikan, O. Penc, C. Peng, H. Peng, J. Penwell, B. S. Peralva, M. M. Perego, D. V. Perepelitsa, E. Perez Codina, L. Perini, H. Pernegger, S. Perrella, R. Peschke, V. D. Peshekhonov, K. Peters, R. F. Y. Peters, B. A. Petersen, T. C. Petersen, E. Petit, A. Petridis, C. Petridou, P. Petroff, E. Petrolo, M. Petrov, F. Petrucci, N. E. Pettersson, A. Peyaud, R. Pezoa, P. W. Phillips, G. Piacquadio, E. Pianori, A. Picazio, E. Piccaro, M. Piccinini, M. A. Pickering, R. Piegaia, J. E. Pilcher, A. D. Pilkington, A. W. J. Pin, M. Pinamonti, J. L. Pinfold, A. Pingel, S. Pires, H. Pirumov, M. Pitt, L. Plazak, M.-A. Pleier, V. Pleskot, E. Plotnikova, P. Plucinski, D. Pluth, R. Poettgen, L. Poggioli, D. Pohl, G. Polesello, A. Poley, A. Policicchio, R. Polifka, A. Polini, C. S. Pollard, V. Polychronakos, K. Pommès, L. Pontecorvo, B. G. Pope, G. A. Popeneciu, D. S. Popovic, A. Poppleton, S. Pospisil, K. Potamianos, I. N. Potrap, C. J. Potter, C. T. Potter, G. Poulard, J. Poveda, V. Pozdnyakov, M. E. Pozo Astigarraga, P. Pralavorio, A. Pranko, S. Prell, D. Price, L. E. Price, M. Primavera, S. Prince, M. Proissl, K. Prokofiev, F. Prokoshin, S. Protopopescu, J. Proudfoot, M. Przybycien, D. Puddu, M. Purohit, P. Puzo, J. Qian, G. Qin, Y. Qin, A. Quadt, W. B. Quayle, M. Queitsch-Maitland, D. Quilty, S. Raddum, V. Radeka, V. Radescu, S. K. Radhakrishnan, P. Radloff, P. Rados, F. Ragusa, G. Rahal, J. A. Raine, S. Rajagopalan, M. Rammensee, C. Rangel-Smith, M. G. Ratti, F. Rauscher, S. Rave, T. Ravenscroft, I. Ravinovich, M. Raymond, A. L. Read, N. P. Readioff, M. Reale, D. M. Rebuzzi, A. Redelbach, G. Redlinger, R. Reece, K. Reeves, L. Rehnisch, J. Reichert, H. Reisin, C. Rembser, H. Ren, M. Rescigno, S. Resconi, O. L. Rezanova, P. Reznicek, R. Rezvani, R. Richter, S. Richter, E. Richter-Was, O. Ricken, M. Ridel, P. Rieck, C. J. Riegel, J. Rieger, O. Rifki, M. Rijssenbeek, A. Rimoldi, M. Rimoldi, L. Rinaldi, B. Ristić, E. Ritsch, I. Riu, F. Rizatdinova, E. Rizvi, C. Rizzi, S. H. Robertson, A. Robichaud-Veronneau, D. Robinson, J. E. M. Robinson, A. Robson, C. Roda, Y. Rodina, A. Rodriguez Perez, D. Rodriguez Rodriguez, S. Roe, C. S. Rogan, O. Røhne, A. Romaniouk, M. Romano, S. M. Romano Saez, E. Romero Adam, N. Rompotis, M. Ronzani, L. Roos, E. Ros, S. Rosati, K. Rosbach, P. Rose, O. Rosenthal, N.-A. Rosien, V. Rossetti, E. Rossi, L. P. Rossi, J. H. N. Rosten, R. Rosten, M. Rotaru, I. Roth, J. Rothberg, D. Rousseau, C. R. Royon, A. Rozanov, Y. Rozen, X. Ruan, F. Rubbo, M. S. Rudolph, F. Rühr, A. Ruiz-Martinez, Z. Rurikova, N. A. Rusakovich, A. Ruschke, H. L. Russell, J. P. Rutherfoord, N. Ruthmann, Y. F. Ryabov, M. Rybar, G. Rybkin, S. Ryu, A. Ryzhov, G. F. Rzehorz, A. F. Saavedra, G. Sabato, S. Sacerdoti, H. F.-W. Sadrozinski, R. Sadykov, F. Safai Tehrani, P. Saha, M. Sahinsoy, M. Saimpert, T. Saito, H. Sakamoto, Y. Sakurai, G. Salamanna, A. Salamon, J. E. Salazar Loyola, D. Salek, P. H. Sales De Bruin, D. Salihagic, A. Salnikov, J. Salt, D. Salvatore, F. Salvatore, A. Salvucci, A. Salzburger, D. Sammel, D. Sampsonidis, J. Sánchez, V. Sanchez Martinez, A. Sanchez Pineda, H. Sandaker, R. L. Sandbach, H. G. Sander, M. Sandhoff, C. Sandoval, R. Sandstroem, D. P. C. Sankey, M. Sannino, A. Sansoni, C. Santoni, R. Santonico, H. Santos, I. Santoyo Castillo, K. Sapp, A. Sapronov, J. G. Saraiva, B. Sarrazin, O. Sasaki, Y. Sasaki, K. Sato, G. Sauvage, E. Sauvan, G. Savage, P. Savard, C. Sawyer, L. Sawyer, J. Saxon, C. Sbarra, A. Sbrizzi, T. Scanlon, D. A. Scannicchio, M. Scarcella, V. Scarfone, J. Schaarschmidt, P. Schacht, B. M. Schachtner, D. Schaefer, R. Schaefer, J. Schaeffer, S. Schaepe, S. Schaetzel, U. Schäfer, A. C. Schaffer, D. Schaile, R. D. Schamberger, V. Scharf, V. A. Schegelsky, D. Scheirich, M. Schernau, C. Schiavi, S. Schier, C. Schillo, M. Schioppa, S. Schlenker, K. R. Schmidt-Sommerfeld, K. Schmieden, C. Schmitt, S. Schmitt, S. Schmitz, B. Schneider, U. Schnoor, L. Schoeffel, A. Schoening, B. D. Schoenrock, E. Schopf, M. Schott, J. Schovancova, S. Schramm, M. Schreyer, N. Schuh, A. Schulte, M. J. Schultens, H.-C. Schultz-Coulon, H. Schulz, M. Schumacher, B. A. Schumm, Ph. Schune, A. Schwartzman, T. A. Schwarz, Ph. Schwegler, H. Schweiger, Ph. Schwemling, R. Schwienhorst, J. Schwindling, T. Schwindt, G. Sciolla, F. Scuri, F. Scutti, J. Searcy, P. Seema, S. C. Seidel, A. Seiden, F. Seifert, J. M. Seixas, G. Sekhniaidze, K. Sekhon, S. J. Sekula, D. M. Seliverstov, N. Semprini-Cesari, C. Serfon, L. Serin, L. Serkin, M. Sessa, R. Seuster, H. Severini, T. Sfiligoj, F. Sforza, A. Sfyrla, E. Shabalina, N. W. Shaikh, L. Y. Shan, R. Shang, J. T. Shank, M. Shapiro, P. B. Shatalov, K. Shaw, S. M. Shaw, A. Shcherbakova, C. Y. Shehu, P. Sherwood, L. Shi, S. Shimizu, C. O. Shimmin, M. Shimojima, M. Shiyakova, A. Shmeleva, D. Shoaleh Saadi, M. J. Shochet, S. Shojaii, S. Shrestha, E. Shulga, M. A. Shupe, P. Sicho, A. M. Sickles, P. E. Sidebo, O. Sidiropoulou, D. Sidorov, A. Sidoti, F. Siegert, Dj. Sijacki, J. Silva, S. B. Silverstein, V. Simak, O. Simard, Lj. Simic, S. Simion, E. Simioni, B. Simmons, D. Simon, M. Simon, P. Sinervo, N. B. Sinev, M. Sioli, G. Siragusa, S. Yu. Sivoklokov, J. Sjölin, M. B. Skinner, H. P. Skottowe, P. Skubic, M. Slater, T. Slavicek, M. Slawinska, K. Sliwa, R. Slovak, V. Smakhtin, B. H. Smart, L. Smestad, J. Smiesko, S. Yu. Smirnov, Y. Smirnov, L. N. Smirnova, O. Smirnova, M. N. K. Smith, R. W. Smith, M. Smizanska, K. Smolek, A. A. Snesarev, S. Snyder, R. Sobie, F. Socher, A. Soffer, D. A. Soh, G. Sokhrannyi, C. A. Solans Sanchez, M. Solar, E. Yu. Soldatov, U. Soldevila, A. A. Solodkov, A. Soloshenko, O. V. Solovyanov, V. Solovyev, P. Sommer, H. Son, H. Y. Song, A. Sood, A. Sopczak, V. Sopko, V. Sorin, D. Sosa, C. L. Sotiropoulou, R. Soualah, A. M. Soukharev, D. South, B. C. Sowden, S. Spagnolo, M. Spalla, M. Spangenberg, F. Spanò, D. Sperlich, F. Spettel, R. Spighi, G. Spigo, L. A. Spiller, M. Spousta, R. D. St. Denis, A. Stabile, R. Stamen, S. Stamm, E. Stanecka, R. W. Stanek, C. Stanescu, M. Stanescu-Bellu, M. M. Stanitzki, S. Stapnes, E. A. Starchenko, G. H. Stark, J. Stark, S. H Stark, P. Staroba, P. Starovoitov, S. Stärz, R. Staszewski, P. Steinberg, B. Stelzer, H. J. Stelzer, O. Stelzer-Chilton, H. Stenzel, G. A. Stewart, J. A. Stillings, M. C. Stockton, M. Stoebe, G. Stoicea, P. Stolte, S. Stonjek, A. R. Stradling, A. Straessner, M. E. Stramaglia, J. Strandberg, S. Strandberg, A. Strandlie, M. Strauss, P. Strizenec, R. Ströhmer, D. M. Strom, R. Stroynowski, A. Strubig, S. A. Stucci, B. Stugu, N. A. Styles, D. Su, J. Su, S. Suchek, Y. Sugaya, M. Suk, V. V. Sulin, S. Sultansoy, T. Sumida, S. Sun, X. Sun, J. E. Sundermann, K. Suruliz, G. Susinno, M. R. Sutton, S. Suzuki, M. Svatos, M. Swiatlowski, I. Sykora, T. Sykora, D. Ta, C. Taccini, K. Tackmann, J. Taenzer, A. Taffard, R. Tafirout, N. Taiblum, H. Takai, R. Takashima, T. Takeshita, Y. Takubo, M. Talby, A. A. Talyshev, K. G. Tan, J. Tanaka, R. Tanaka, S. Tanaka, B. B. Tannenwald, S. Tapia Araya, S. Tapprogge, S. Tarem, G. F. Tartarelli, P. Tas, M. Tasevsky, T. Tashiro, E. Tassi, A. Tavares Delgado, Y. Tayalati, A. C. Taylor, G. N. Taylor, P. T. E. Taylor, W. Taylor, F. A. Teischinger, P. Teixeira-Dias, D. Temple, H. Ten Kate, P. K. Teng, J. J. Teoh, F. Tepel, S. Terada, K. Terashi, J. Terron, S. Terzo, M. Testa, R. J. Teuscher, T. Theveneaux-Pelzer, J. P. Thomas, J. Thomas-Wilsker, E. N. Thompson, P. D. Thompson, A. S. Thompson, L. A. Thomsen, E. Thomson, M. Thomson, M. J. Tibbetts, R. E. Ticse Torres, V. O. Tikhomirov, Yu. A. Tikhonov, S. Timoshenko, P. Tipton, S. Tisserant, K. Todome, T. Todorov, S. Todorova-Nova, J. Tojo, S. Tokár, K. Tokushuku, E. Tolley, L. Tomlinson, M. Tomoto, L. Tompkins, K. Toms, B. Tong, E. Torrence, H. Torres, E. Torró Pastor, J. Toth, F. Touchard, D. R. Tovey, T. Trefzger, A. Tricoli, I. M. Trigger, S. Trincaz-Duvoid, M. F. Tripiana, W. Trischuk, B. Trocmé, A. Trofymov, C. Troncon, M. Trottier-McDonald, M. Trovatelli, L. Truong, M. Trzebinski, A. Trzupek, J. C.-L. Tseng, P. V. Tsiareshka, G. Tsipolitis, N. Tsirintanis, S. Tsiskaridze, V. Tsiskaridze, E. G. Tskhadadze, K. M. Tsui, I. I. Tsukerman, V. Tsulaia, S. Tsuno, D. Tsybychev, A. Tudorache, V. Tudorache, A. N. Tuna, S. A. Tupputi, S. Turchikhin, D. Turecek, D. Turgeman, R. Turra, A. J. Turvey, P. M. Tuts, M. Tyndel, G. Ucchielli, I. Ueda, M. Ughetto, F. Ukegawa, G. Unal, A. Undrus, G. Unel, F. C. Ungaro, Y. Unno, C. Unverdorben, J. Urban, P. Urquijo, P. Urrejola, G. Usai, A. Usanova, L. Vacavant, V. Vacek, B. Vachon, C. Valderanis, E. Valdes Santurio, N. Valencic, S. Valentinetti, A. Valero, L. Valéry, S. Valkar, S. Vallecorsa, J. A. Valls Ferrer, W. Van Den Wollenberg, P. C. Van Der Deijl, R. van der Geer, H. van der Graaf, N. van Eldik, P. van Gemmeren, J. Van Nieuwkoop, I. van Vulpen, M. C. van Woerden, M. Vanadia, W. Vandelli, R. Vanguri, A. Vaniachine, P. Vankov, G. Vardanyan, R. Vari, E. W. Varnes, T. Varol, D. Varouchas, A. Vartapetian, K. E. Varvell, J. G. Vasquez, F. Vazeille, T. Vazquez Schroeder, J. Veatch, L. M. Veloce, F. Veloso, S. Veneziano, A. Ventura, M. Venturi, N. Venturi, A. Venturini, V. Vercesi, M. Verducci, W. Verkerke, J. C. Vermeulen, A. Vest, M. C. Vetterli, O. Viazlo, I. Vichou, T. Vickey, O. E. Vickey Boeriu, G. H. A. Viehhauser, S. Viel, L. Vigani, R. Vigne, M. Villa, M. Villaplana Perez, E. Vilucchi, M. G. Vincter, V. B. Vinogradov, C. Vittori, I. Vivarelli, S. Vlachos, M. Vlasak, M. Vogel, P. Vokac, G. Volpi, M. Volpi, H. von der Schmitt, E. von Toerne, V. Vorobel, K. Vorobev, M. Vos, R. Voss, J. H. Vossebeld, N. Vranjes, M. Vranjes Milosavljevic, V. Vrba, M. Vreeswijk, R. Vuillermet, I. Vukotic, Z. Vykydal, P. Wagner, W. Wagner, H. Wahlberg, S. Wahrmund, J. Wakabayashi, J. Walder, R. Walker, W. Walkowiak, V. Wallangen, C. Wang, C. Wang, F. Wang, H. Wang, H. Wang, J. Wang, J. Wang, K. Wang, R. Wang, S. M. Wang, T. Wang, T. Wang, W. Wang, X. Wang, C. Wanotayaroj, A. Warburton, C. P. Ward, D. R. Wardrope, A. Washbrook, P. M. Watkins, A. T. Watson, M. F. Watson, G. Watts, S. Watts, B. M. Waugh, S. Webb, M. S. Weber, S. W. Weber, J. S. Webster, A. R. Weidberg, B. Weinert, J. Weingarten, C. Weiser, H. Weits, P. S. Wells, T. Wenaus, T. Wengler, S. Wenig, N. Wermes, M. Werner, M. D. Werner, P. Werner, M. Wessels, J. Wetter, K. Whalen, N. L. Whallon, A. M. Wharton, A. White, M. J. White, R. White, D. Whiteson, F. J. Wickens, W. Wiedenmann, M. Wielers, P. Wienemann, C. Wiglesworth, L. A. M. Wiik-Fuchs, A. Wildauer, F. Wilk, H. G. Wilkens, H. H. Williams, S. Williams, C. Willis, S. Willocq, J. A. Wilson, I. Wingerter-Seez, F. Winklmeier, O. J. Winston, B. T. Winter, M. Wittgen, J. Wittkowski, M. W. Wolter, H. Wolters, S. D. Worm, B. K. Wosiek, J. Wotschack, M. J. Woudstra, K. W. Wozniak, M. Wu, M. Wu, S. L. Wu, X. Wu, Y. Wu, T. R. Wyatt, B. M. Wynne, S. Xella, D. Xu, L. Xu, B. Yabsley, S. Yacoob, R. Yakabe, D. Yamaguchi, Y. Yamaguchi, A. Yamamoto, S. Yamamoto, T. Yamanaka, K. Yamauchi, Y. Yamazaki, Z. Yan, H. Yang, H. Yang, Y. Yang, Z. Yang, W.-M. Yao, Y. C. Yap, Y. Yasu, E. Yatsenko, K. H. Yau Wong, J. Ye, S. Ye, I. Yeletskikh, A. L. Yen, E. Yildirim, K. Yorita, R. Yoshida, K. Yoshihara, C. Young, C. J. S. Young, S. Youssef, D. R. Yu, J. Yu, J. M. Yu, J. Yu, L. Yuan, S. P. Y. Yuen, I. Yusuff, B. Zabinski, R. Zaidan, A. M. Zaitsev, N. Zakharchuk, J. Zalieckas, A. Zaman, S. Zambito, L. Zanello, D. Zanzi, C. Zeitnitz, M. Zeman, A. Zemla, J. C. Zeng, Q. Zeng, K. Zengel, O. Zenin, T. Ženiš, D. Zerwas, D. Zhang, F. Zhang, G. Zhang, H. Zhang, J. Zhang, L. Zhang, R. Zhang, R. Zhang, X. Zhang, Z. Zhang, X. Zhao, Y. Zhao, Z. Zhao, A. Zhemchugov, J. Zhong, B. Zhou, C. Zhou, L. Zhou, L. Zhou, M. Zhou, N. Zhou, C. G. Zhu, H. Zhu, J. Zhu, Y. Zhu, X. Zhuang, K. Zhukov, A. Zibell, D. Zieminska, N. I. Zimine, C. Zimmermann, S. Zimmermann, Z. Zinonos, M. Zinser, M. Ziolkowski, L. Živković, G. Zobernig, A. Zoccoli, M. zur Nedden, L. Zwalinski, ATLAS Collaboration

**Affiliations:** 1 Department of Physics, University of Adelaide, Adelaide, Australia; 2 Physics Department, SUNY Albany, Albany, NY USA; 3 Department of Physics, University of Alberta, Edmonton, AB Canada; 4 Department of Physics, Ankara University, Ankara, Turkey; 5 Istanbul Aydin University, Istanbul, Turkey; 6 Division of Physics, TOBB University of Economics and Technology, Ankara, Turkey; 7 LAPP, CNRS/IN2P3 and Université Savoie Mont Blanc, 00, France; 8 High Energy Physics Division, Argonne National Laboratory, Argonne, IL USA; 9 Department of Physics, University of Arizona, Tucson, AZ USA; 10 Department of Physics, The University of Texas at Arlington, Arlington, TX USA; 11 Physics Department, National and Kapodistrian University of Athens, Athens, Greece; 12 Physics Department, National Technical University of Athens, Zografou, Greece; 13 Department of Physics, The University of Texas at Austin, Austin, TX USA; 14 Institute of Physics, Azerbaijan Academy of Sciences, Baku, Azerbaijan; 15 Institut de Física d’Altes Energies (IFAE), The Barcelona Institute of Science and Technology, Barcelona, Spain; 16 Institute of Physics, University of Belgrade, Belgrade, Serbia; 17 Department for Physics and Technology, University of Bergen, Bergen, Norway; 18 Physics Division, Lawrence Berkeley National Laboratory and University of California, Berkeley, CA USA; 19 Department of Physics, Humboldt University, Berlin, Germany; 20 Albert Einstein Center for Fundamental Physics and Laboratory for High Energy Physics, University of Bern, Bern, Switzerland; 21 School of Physics and Astronomy, University of Birmingham, Birmingham, UK; 22 Department of Physics, Bogazici University, Istanbul, Turkey; 23 Department of Physics Engineering, Gaziantep University, Gaziantep, Turkey; 24 Faculty of Engineering and Natural Sciences, Istanbul Bilgi University, Istanbul, Turkey; 25 Faculty of Engineering and Natural Sciences, Bahcesehir University, Istanbul, Turkey; 26 Centro de Investigaciones, Universidad Antonio Narino, Bogotá, Colombia; 27 INFN Sezione di Bologna, Bologna, Italy; 28 Dipartimento di Fisica e Astronomia, Università di Bologna, Bologna, Italy; 29 Physikalisches Institut, University of Bonn, Bonn, Germany; 30 Department of Physics, Boston University, Boston, MA USA; 31 Department of Physics, Brandeis University, Waltham, MA USA; 32 Universidade Federal do Rio De Janeiro COPPE/EE/IF, Rio de Janeiro, Brazil; 33 Electrical Circuits Department, Federal University of Juiz de Fora (UFJF), Juiz de Fora, Brazil; 34 Federal University of Sao Joao del Rei (UFSJ), Sao Joao del Rei, Brazil; 35 Instituto de Fisica, Universidade de Sao Paulo, Sao Paulo, Brazil; 36 Physics Department, Brookhaven National Laboratory, Upton, NY USA; 37 Transilvania University of Brasov, Brasov, Romania; 38 Horia Hulubei National Institute of Physics and Nuclear Engineering, Bucharest, Romania; 39 Physics Department, National Institute for Research and Development of Isotopic and Molecular Technologies, Cluj-Napoca, Romania; 40 University Politehnica Bucharest, Bucharest, Romania; 41 West University in Timisoara, Timisoara, Romania; 42 Departamento de Física, Universidad de Buenos Aires, Buenos Aires, Argentina; 43 Cavendish Laboratory, University of Cambridge, Cambridge, UK; 44 Department of Physics, Carleton University, Ottawa, ON Canada; 45 CERN, Geneva, Switzerland; 46 Enrico Fermi Institute, University of Chicago, Chicago, IL USA; 47 Departamento de Física, Pontificia Universidad Católica de Chile, Santiago, Chile; 48 Departamento de Física, Universidad Técnica Federico Santa María, Valparaiso, Chile; 49 Institute of High Energy Physics, Chinese Academy of Sciences, Beijing, China; 50 Department of Physics, Nanjing University, Jiangsu, China; 51 Physics Department, Tsinghua University, Beijing, 100084 China; 52 Université Clermont Auvergne, CNRS/IN2P3, LPC, Clermont-Ferrand, France; 53 Nevis Laboratory, Columbia University, Irvington, NY USA; 54 Niels Bohr Institute, University of Copenhagen, Kobenhavn, Denmark; 55 INFN Gruppo Collegato di Cosenza, Laboratori Nazionali di Frascati, Frascati, Italy; 56 Dipartimento di Fisica, Università della Calabria, Rende, Italy; 57 Faculty of Physics and Applied Computer Science, AGH University of Science and Technology, Kraków, Poland; 58 Marian Smoluchowski Institute of Physics, Jagiellonian University, Kraków, Poland; 59 Institute of Nuclear Physics Polish Academy of Sciences, Kraków, Poland; 60 Physics Department, Southern Methodist University, Dallas, TX USA; 61 Physics Department, University of Texas at Dallas, Richardson, TX USA; 62 DESY, Hamburg and Zeuthen, Germany; 63 Lehrstuhl für Experimentelle Physik IV, Technische Universität Dortmund, Dortmund, Germany; 64 Institut für Kern- und Teilchenphysik, Technische Universität Dresden, Dresden, Germany; 65 Department of Physics, Duke University, Durham, NC USA; 66 SUPA-School of Physics and Astronomy, University of Edinburgh, Edinburgh, UK; 67 INFN e Laboratori Nazionali di Frascati, Frascati, Italy; 68 Fakultät für Mathematik und Physik, Albert-Ludwigs-Universität, Freiburg, Germany; 69 Departement de Physique Nucleaire et Corpusculaire, Université de Genève, Geneva, Switzerland; 70 INFN Sezione di Genova, Genoa, Italy; 71 Dipartimento di Fisica, Università di Genova, Genoa, Italy; 72 E. Andronikashvili Institute of Physics, Iv. Javakhishvili Tbilisi State University, Tbilisi, Georgia; 73 High Energy Physics Institute, Tbilisi State University, Tbilisi, Georgia; 74 II Physikalisches Institut, Justus-Liebig-Universität Giessen, Giessen, Germany; 75 SUPA-School of Physics and Astronomy, University of Glasgow, Glasgow, UK; 76 II Physikalisches Institut, Georg-August-Universität, Göttingen, Germany; 77 Laboratoire de Physique Subatomique et de Cosmologie, Université Grenoble-Alpes, CNRS/IN2P3, Grenoble, France; 78 Laboratory for Particle Physics and Cosmology, Harvard University, Cambridge, MA USA; 79 Department of Modern Physics and State Key Laboratory of Particle Detection and Electronics, University of Science and Technology of China, Anhui, China; 80 Kirchhoff-Institut für Physik, Ruprecht-Karls-Universität Heidelberg, Heidelberg, Germany; 81 Physikalisches Institut, Ruprecht-Karls-Universität Heidelberg, Heidelberg, Germany; 82 ZITI Institut für technische Informatik, Ruprecht-Karls-Universität Heidelberg, Mannheim, Germany; 83 Faculty of Applied Information Science, Hiroshima Institute of Technology, Hiroshima, Japan; 84 Department of Physics, The Chinese University of Hong Kong, Shatin, N.T. Hong Kong; 85 Department of Physics, The University of Hong Kong, Hong Kong, China; 86 Department of Physics and Institute for Advanced Study, The Hong Kong University of Science and Technology, Clear Water Bay, Kowloon, Hong Kong, China; 87 Department of Physics, Indiana University, Bloomington, IN USA; 88 Institut für Astro- und Teilchenphysik, Leopold-Franzens-Universität, Innsbruck, Austria; 89 University of Iowa, Iowa City, IA USA; 90 Department of Physics and Astronomy, Iowa State University, Ames, IA USA; 91 Joint Institute for Nuclear Research, JINR Dubna, Dubna, Russia; 92 KEK, High Energy Accelerator Research Organization, Tsukuba, Japan; 93 Graduate School of Science, Kobe University, Kobe, Japan; 94 Faculty of Science, Kyoto University, Kyoto, Japan; 95 Kyoto University of Education, Kyoto, Japan; 96 Research Center for Advanced Particle Physics and Department of Physics, Kyushu University, Fukuoka, Japan; 97 Instituto de Física La Plata, Universidad Nacional de La Plata and CONICET, La Plata, Argentina; 98 Physics Department, Lancaster University, Lancaster, UK; 99 INFN Sezione di Lecce, Lecce, Italy; 100 Dipartimento di Matematica e Fisica, Università del Salento, Lecce, Italy; 101 Oliver Lodge Laboratory, University of Liverpool, Liverpool, UK; 102 Department of Experimental Particle Physics, Jožef Stefan Institute and Department of Physics, University of Ljubljana, Ljubljana, Slovenia; 103 School of Physics and Astronomy, Queen Mary University of London, London, UK; 104 Department of Physics, Royal Holloway University of London, Surrey, UK; 105 Department of Physics and Astronomy, University College London, London, UK; 106 Louisiana Tech University, Ruston, LA USA; 107 Laboratoire de Physique Nucléaire et de Hautes Energies, UPMC and Université Paris-Diderot and CNRS/IN2P3, Paris, France; 108 Fysiska institutionen, Lunds universitet, Lund, Sweden; 109 Departamento de Fisica Teorica C-15, Universidad Autonoma de Madrid, Madrid, Spain; 110 Institut für Physik, Universität Mainz, Mainz, Germany; 111 School of Physics and Astronomy, University of Manchester, Manchester, UK; 112 CPPM, Aix-Marseille Université and CNRS/IN2P3, Marseille, France; 113 Department of Physics, University of Massachusetts, Amherst, MA USA; 114 Department of Physics, McGill University, Montreal, QC Canada; 115 School of Physics, University of Melbourne, Victoria, Australia; 116 Department of Physics, The University of Michigan, Ann Arbor, MI USA; 117 Department of Physics and Astronomy, Michigan State University, East Lansing, MI USA; 118 INFN Sezione di Milano, Milan, Italy; 119 Dipartimento di Fisica, Università di Milano, Milan, Italy; 120 B.I. Stepanov Institute of Physics, National Academy of Sciences of Belarus, Minsk, Republic of Belarus; 121 Research Institute for Nuclear Problems of Byelorussian State University, Minsk, Republic of Belarus; 122 Group of Particle Physics, University of Montreal, Montreal, QC Canada; 123 P.N. Lebedev Physical Institute of the Russian Academy of Sciences, Moscow, Russia; 124 Institute for Theoretical and Experimental Physics (ITEP), Moscow, Russia; 125 National Research Nuclear University MEPhI, Moscow, Russia; 126 D.V. Skobeltsyn Institute of Nuclear Physics, M.V. Lomonosov Moscow State University, Moscow, Russia; 127 Fakultät für Physik, Ludwig-Maximilians-Universität München, Munich, Germany; 128 Max-Planck-Institut für Physik (Werner-Heisenberg-Institut), Munich, Germany; 129 Nagasaki Institute of Applied Science, Nagasaki, Japan; 130 Graduate School of Science and Kobayashi-Maskawa Institute, Nagoya University, Nagoya, Japan; 131 INFN Sezione di Napoli, Naples, Italy; 132 Dipartimento di Fisica, Università di Napoli, Naples, Italy; 133 Department of Physics and Astronomy, University of New Mexico, Albuquerque, NM USA; 134 Institute for Mathematics, Astrophysics and Particle Physics, Radboud University Nijmegen/Nikhef, Nijmegen, The Netherlands; 135 Nikhef National Institute for Subatomic Physics and University of Amsterdam, Amsterdam, The Netherlands; 136 Department of Physics, Northern Illinois University, DeKalb, IL USA; 137 Budker Institute of Nuclear Physics, SB RAS, Novosibirsk, Russia; 138 Department of Physics, New York University, New York, NY USA; 139 Ohio State University, Columbus, OH USA; 140 Faculty of Science, Okayama University, Okayama, Japan; 141 Homer L. Dodge Department of Physics and Astronomy, University of Oklahoma, Norman, OK USA; 142 Department of Physics, Oklahoma State University, Stillwater, OK USA; 143 Palacký University, RCPTM, Olomouc, Czech Republic; 144 Center for High Energy Physics, University of Oregon, Eugene, OR USA; 145 LAL, Univ. Paris-Sud, CNRS/IN2P3, Université Paris-Saclay, Orsay, France; 146 Graduate School of Science, Osaka University, Osaka, Japan; 147 Department of Physics, University of Oslo, Oslo, Norway; 148 Department of Physics, Oxford University, Oxford, UK; 149 INFN Sezione di Pavia, Pavia, Italy; 150 Dipartimento di Fisica, Università di Pavia, Pavia, Italy; 151 Department of Physics, University of Pennsylvania, Philadelphia, PA USA; 152 National Research Centre “Kurchatov Institute” B.P. Konstantinov Petersburg Nuclear Physics Institute, St. Petersburg, Russia; 153 INFN Sezione di Pisa, Pisa, Italy; 154 Dipartimento di Fisica E. Fermi, Università di Pisa, Pisa, Italy; 155 Department of Physics and Astronomy, University of Pittsburgh, Pittsburgh, PA USA; 156 Laboratório de Instrumentação e Física Experimental de Partículas-LIP, Lisbon, Portugal; 157 Faculdade de Ciências, Universidade de Lisboa, Lisbon, Portugal; 158 Department of Physics, University of Coimbra, Coimbra, Portugal; 159 Centro de Física Nuclear da Universidade de Lisboa, Lisbon, Portugal; 160 Departamento de Fisica, Universidade do Minho, Braga, Portugal; 161 Departamento de Fisica Teorica y del Cosmos and CAFPE, Universidad de Granada, Granada, Spain; 162 Dep Fisica and CEFITEC of Faculdade de Ciencias e Tecnologia, Universidade Nova de Lisboa, Caparica, Portugal; 163 Institute of Physics, Academy of Sciences of the Czech Republic, Prague, Czech Republic; 164 Czech Technical University in Prague, Prague, Czech Republic; 165 Charles University, Faculty of Mathematics and Physics, Prague, Czech Republic; 166 State Research Center Institute for High Energy Physics (Protvino), NRC KI, Protvino, Russia; 167 Particle Physics Department, Rutherford Appleton Laboratory, Didcot, UK; 168 INFN Sezione di Roma, Rome, Italy; 169 Dipartimento di Fisica, Sapienza Università di Roma, Rome, Italy; 170 INFN Sezione di Roma Tor Vergata, Rome, Italy; 171 Dipartimento di Fisica, Università di Roma Tor Vergata, Rome, Italy; 172 INFN Sezione di Roma Tre, Rome, Italy; 173 Dipartimento di Matematica e Fisica, Università Roma Tre, Rome, Italy; 174 Faculté des Sciences Ain Chock, Réseau Universitaire de Physique des Hautes Energies-Université Hassan II, Casablanca, Morocco; 175 Centre National de l’Energie des Sciences Techniques Nucleaires, Rabat, Morocco; 176 Faculté des Sciences Semlalia, Université Cadi Ayyad, LPHEA-Marrakech, Marrakech, Morocco; 177 Faculté des Sciences, Université Mohamed Premier and LPTPM, Oujda, Morocco; 178 Faculté des Sciences, Université Mohammed V, Rabat, Morocco; 179 DSM/IRFU (Institut de Recherches sur les Lois Fondamentales de l’Univers), CEA Saclay (Commissariat à l’Energie Atomique et aux Energies Alternatives), Gif-sur-Yvette, France; 180 Santa Cruz Institute for Particle Physics, University of California Santa Cruz, Santa Cruz, CA USA; 181 Department of Physics, University of Washington, Seattle, WA USA; 182 School of Physics, Shandong University, Shandong, China; 183 Department of Physics and Astronomy, Key Laboratory for Particle Physics, Astrophysics and Cosmology, Ministry of Education; Shanghai Key Laboratory for Particle Physics and Cosmology, Shanghai Jiao Tong University; (Also at PKU-CHEP), Shanghai, China; 184 Department of Physics and Astronomy, University of Sheffield, Sheffield, UK; 185 Department of Physics, Shinshu University, Nagano, Japan; 186 FDepartment Physik, Universität Siegen, Siegen, Germany; 187 Department of Physics, Simon Fraser University, Burnaby, BC Canada; 188 SLAC National Accelerator Laboratory, Stanford, CA USA; 189 Faculty of Mathematics, Physics and Informatics, Comenius University, Bratislava, Slovak Republic; 190 Department of Subnuclear Physics, Institute of Experimental Physics of the Slovak Academy of Sciences, Kosice, Slovak Republic; 191 Department of Physics, University of Cape Town, Cape Town, South Africa; 192 Department of Physics, University of Johannesburg, Johannesburg, South Africa; 193 School of Physics, University of the Witwatersrand, Johannesburg, South Africa; 194 Department of Physics, Stockholm University, Stockholm, Sweden; 195 The Oskar Klein Centre, Stockholm, Sweden; 196 Physics Department, Royal Institute of Technology, Stockholm, Sweden; 197 Departments of Physics and Astronomy and Chemistry, Stony Brook University, Stony Brook, NY USA; 198 Department of Physics and Astronomy, University of Sussex, Brighton, UK; 199 School of Physics, University of Sydney, Sydney, Australia; 200 Institute of Physics, Academia Sinica, Taipei, Taiwan; 201 Department of Physics, Technion: Israel Institute of Technology, Haifa, Israel; 202 Raymond and Beverly Sackler School of Physics and Astronomy, Tel Aviv University, Tel Aviv, Israel; 203 Department of Physics, Aristotle University of Thessaloniki, Thessaloniki, Greece; 204 International Center for Elementary Particle Physics and Department of Physics, The University of Tokyo, Tokyo, Japan; 205 Graduate School of Science and Technology, Tokyo Metropolitan University, Tokyo, Japan; 206 Department of Physics, Tokyo Institute of Technology, Tokyo, Japan; 207 Tomsk State University, Tomsk, Russia; 208 Department of Physics, University of Toronto, Toronto, ON Canada; 209 TRIUMF, Vancouver, BC Canada; 210 Department of Physics and Astronomy, York University, Toronto, ON Canada; 211 Faculty of Pure and Applied Sciences, and Center for Integrated Research in Fundamental Science and Engineering, University of Tsukuba, Tsukuba, Japan; 212 Department of Physics and Astronomy, Tufts University, Medford, MA USA; 213 Department of Physics and Astronomy, University of California Irvine, Irvine, CA USA; 214 INFN Gruppo Collegato di Udine, Sezione di Trieste, Udine, Italy; 215 ICTP, Trieste, Italy; 216 Dipartimento di Chimica, Fisica e Ambiente, Università di Udine, Udine, Italy; 217 Department of Physics and Astronomy, University of Uppsala, Uppsala, Sweden; 218 Department of Physics, University of Illinois, Urbana, IL USA; 219 Instituto de Fisica Corpuscular (IFIC), Centro Mixto Universidad de Valencia-CSIC, Valencia, Spain; 220 Department of Physics, University of British Columbia, Vancouver, BC Canada; 221 Department of Physics and Astronomy, University of Victoria, Victoria, BC Canada; 222 Department of Physics, University of Warwick, Coventry, UK; 223 Waseda University, Tokyo, Japan; 224 Department of Particle Physics, The Weizmann Institute of Science, Rehovot, Israel; 225 Department of Physics, University of Wisconsin, Madison, WI USA; 226 Fakultät für Physik und Astronomie, Julius-Maximilians-Universität, Würzburg, Germany; 227 Fakultät für Mathematik und Naturwissenschaften, Fachgruppe Physik, Bergische Universität Wuppertal, Wuppertal, Germany; 228 Department of Physics, Yale University, New Haven, CT USA; 229 Yerevan Physics Institute, Yerevan, Armenia; 230 Centre de Calcul de l’Institut National de Physique Nucléaire et de Physique des Particules (IN2P3), Villeurbanne, France; 231 CERN, 1211 Geneva 23, Switzerland

## Abstract

This paper presents the method and performance of primary vertex reconstruction in proton–proton collision data recorded by the ATLAS experiment during Run 1 of the LHC. The studies presented focus on data taken during 2012 at a centre-of-mass energy of $\sqrt{s} = 8$ TeV. The performance has been measured as a function of the number of interactions per bunch crossing over a wide range, from one to seventy. The measurement of the position and size of the luminous region and its use as a constraint to improve the primary vertex resolution are discussed. A longitudinal vertex position resolution of about $30\;\upmu {\text {m}}$ is achieved for events with high multiplicity of reconstructed tracks. The transverse position resolution is better than $20\;\upmu {\text {m}}$ and is dominated by the precision on the size of the luminous region. An analytical model is proposed to describe the primary vertex reconstruction efficiency as a function of the number of interactions per bunch crossing and of the longitudinal size of the luminous region. Agreement between the data and the predictions of this model is better than 3% up to seventy interactions per bunch crossing.

## Introduction

Efficient and precise reconstruction of primary vertices, defined as the points in space where proton–proton (*pp*) interactions have occurred, is an important element of data analysis at the LHC. It is of direct relevance to the reconstruction of hard-scatter interactions, in which the correct assignment of charged-particle trajectories to the hard-scatter primary vertex is essential in reconstructing the full kinematic properties of the event. An aspect of primary vertex reconstruction requiring special attention is the superposition of multiple inelastic *pp* interactions reconstructed as a single physics event with many primary vertices. These additional primary vertices, which are usually soft-QCD interactions related to the dominant components of the total cross section, are referred to as pile-up. The average number of inelastic *pp* interactions per bunch crossing under constant beam conditions is denoted as $\mu$ and is directly related to the instantaneous luminosity [1]. The primary vertex reconstruction is also important for the determination of the luminous region, or beam spot, where collisions take place within the ATLAS detector.

This paper describes the performance of primary vertex reconstruction with the ATLAS detector, during Run 1 of the LHC from 2010 to 2012. The studies presented here are based on the data collected in 2012 at a proton–proton centre-of-mass energy $\sqrt{s} = 8$ TeV. Averaged over the 2012 dataset, $\mu$ was approximately 20. The 2012 data are representative of the full set of data taken from 2010 to 2012 in terms of the primary vertex performance. Studies in this paper make use of dedicated datasets recorded at very low values of $\mu$ ($\mu = 0.01$), thereby providing a measurement of the performance in the absence of pile-up. Data recorded with the highest number of interactions per bunch crossing, leading to values of $\mu$ up to 72, are used to study the various mechanisms that lead to a degradation of the primary vertex reconstruction as pile-up increases.

The paper is organised as follows: Sect. 2 provides a brief description of the ATLAS detector, a description of pile-up determination and a discussion of the parameters of the LHC accelerator that determine the size of the luminous region. Section 3 describes the data and Monte Carlo (MC) simulation samples used. Section 4 presents the algorithms for primary vertex reconstruction in ATLAS. The measurement and stability of the beam-spot parameters and their use as a constraint in primary vertex reconstruction are discussed. The predicted impact of pile-up contamination on the reconstruction and selection of primary vertices from hard-scatter processes is discussed in Sect. 5. Studies of single vertex reconstruction in minimum-bias data and the related comparisons to MC simulation are presented in Sect. 6. Section 7 describes the performance of vertex reconstruction in high pile-up conditions. In Sect. 8, the results of studies presented in Sects. 5 through 7 are used to model the efficiency of primary vertex reconstruction in simulation, to predict its behaviour at high pile-up, and to compare the predictions to data. Summary and conclusions are presented in Sect. 9.

## The ATLAS detector and LHC beam parameters

The ATLAS detector [2] is a multi-purpose detector with a cylindrical geometry. It is comprised of an inner detector (ID) surrounded by a thin superconducting solenoid, a calorimeter system and a muon spectrometer embedded in a toroidal magnetic field. The ID is the primary detector used for vertex reconstruction and it is described in further detail below in Sect. 2.1. Outside of the ID and the solenoid are electromagnetic sampling calorimeters made of liquid argon as the active material and lead as an absorber. Surrounding the electromagnetic calorimeter is the iron and scintillator tile calorimeter for hadronic energy measurements. In the forward regions it is complemented by two end-cap calorimeters made of liquid argon and copper or tungsten. The muon spectrometer surrounds the calorimeters and consists of three large superconducting eight-coil toroids, a system of tracking chambers, and detectors for triggering.

### The ATLAS inner detector

**Fig. 1:**
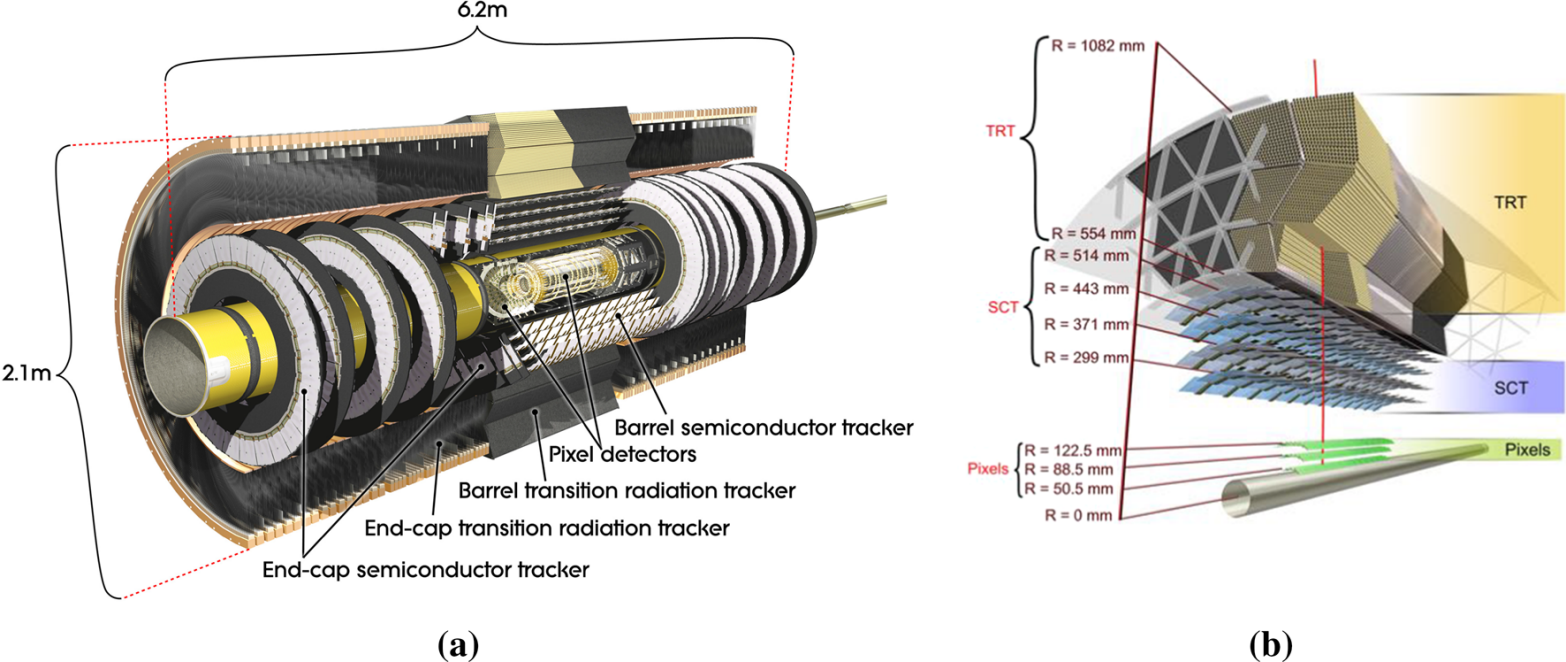
Schematic views of the ATLAS Run 1 inner detector: **a** barrel and end-cap sections; **b** cross section of the barrel section showing the TRT, SCT, and pixel sub-detectors

The inner detector covers the pseudorapidity^1^ range $|\eta |<$ 2.5. Schematic views of the Run 1 inner detector are presented in Fig. 1.

Particle trajectories are identified using the combined information from the sub-detectors of the ID: the innermost silicon pixel detector, the surrounding silicon microstrip semiconductor tracker (SCT), and the transition radiation tracker (TRT), made of straw tubes filled with a Xe-$\text {C}\text {O}_2$ gas mixture [3]. All three sub-systems are divided into a barrel section and two end-caps. The barrel sections consist of several cylindrical layers, while the end-caps are composed of radial disks and wheels. The sensitive regions of the three sub-detectors cover radial distances in the barrel section from 50.5 to 122.5, 299 to 514, and 554 to 1082 mm. Typical position resolutions are 10, 17, and $130 \;\upmu {\text {m}}$ for the transverse coordinate in the pixel detector, the SCT, and the TRT respectively. In the case of the pixel and SCT, the resolutions in the *z*-coordinate are 115 and $580 \;\upmu {\text {m}}$. The superconducting solenoid coil around the tracking system produces a 2 T axial magnetic field. A track from a charged particle traversing the barrel detector would typically have 11 measurements in the silicon detector^2^ (3 pixel clusters and 8 strip clusters) and more than 30 measurements in the TRT [4].

### The minimum-bias trigger

A minimum-bias trigger was used to select the data presented in this paper. This trigger is designed to record a random selection of bunch crossings, unbiased by any hard physics produced in the bunch crossing, by using a signal from the minimum-bias trigger scintillators (MBTS). The MBTS are mounted at each end of the detector in front of the liquid-argon end-cap calorimeter cryostats at $z = \pm 3.56$ m, covering the range $2.09< |\eta | < 3.84$. The MBTS trigger used for this paper requires one hit above threshold from either side of the detector, referred to as a single-arm trigger [4].

### Determination of pile-up interactions

Depending on the length of the read-out window of a sub-detector, signals from neighbouring bunch crossings can be present simultaneously when the detector is read out. The impact of interactions from the neighbouring bunch crossings is referred to as out-of-time pile-up, while in-time pile-up results from the presence of multiple *pp* interactions in the same bunch crossing.

During most of Run 1 of the LHC, the separation of proton bunches was 50 ns. The timing resolution of the inner detector components is about 25 ns. This is sufficient for the out-of-time pile-up to have a much smaller impact on ID measurements than the in-time pile-up. As a consequence the number of reconstructed vertices is a direct measure of the amount of in-time pile-up on an event-by-event basis.

The instantaneous luminosity, *L*, can be expressed in terms of the visible interaction rate, $R^{\text {v}is}_{\text {inel}}$, and the visible inelastic cross section, $\sigma ^{\text {v}is}_{\text {inel}}$, as:

1 $$\begin{aligned} L = \frac{R^{ {{\rm v}}is}_{ {{\rm inel}}}}{\sigma ^{ {{\rm v}}is}_{ {{\rm inel}}}}. \end{aligned}$$

The inelastic cross section, $\sigma _{\text {i}nel}$, and the visible inelastic cross section are related through: $\sigma ^{\text {v}is} _{\text {i}nel}~=~\epsilon \sigma _{\text {i}nel}$. Here $\epsilon$ is the efficiency of the detector to record an inelastic collision. The inelastic cross section is defined as the total cross section minus the elastic cross section.

In practice, the full rate of inelastic collisions is never directly measured. Only a fraction of it is observable in the detector due to the $\eta$ acceptance. The luminosity is measured using a set of dedicated detectors which allow bunch-by-bunch measurements. The luminosity detectors are calibrated using dedicated Van der Meer scans [5]. The uncertainty in the luminosity measurement is 1.9% [1].

The number of *pp* inelastic interactions per bunch crossing follows a Poisson distribution with mean value $\mu$. Assuming that the *pp* collider operates at a revolution frequency $f_{\text {r}}$ with $n_{\text {b}}$ interacting bunches per beam, the luminosity can also be expressed as:

2 $$\begin{aligned} L = \frac{ \mu \; n_{\text {b}} \; f_{\text {r}}}{\sigma _{\text {inel}}}. \end{aligned}$$

The value of $\mu$ changes during data-taking as a function of time: it decreases with decreasing beam intensity and increasing emittance. The highest value is at the start of the stable beam period of the fill. For the studies presented in this paper, $\mu$ is calculated using Eq. (2). The value of the inelastic cross section at 8 TeV centre-of-mass energy is 71.5 mb, taken from the PYTHIA8 MC generator [6]. Experimental measurements [7, 8] are found to be compatible with the cross section predicted by PYTHIA8. The overall uncertainty in $\mu$ is 4%, which is derived from the quadratic sum of the uncertainties in the luminosity and in the inelastic cross section.

### Parameters affecting the luminous region at the LHC

The size, position and shape of the luminous region, or beam spot, are determined by the operating parameters of the beams and magnets of the LHC [9]. The transverse size is determined by the focusing of the LHC beams near the interaction region and by the spread in position–momentum phase space of the protons within the colliding bunches. The latter is quantified by the geometric emittance $\varepsilon _{}$ of the beams, or equivalently by the normalised emittance defined as $\varepsilon _{\text {N} } = \beta _v \, \gamma \, \varepsilon _{}$, where $\beta _v$ and $\gamma$ are the relativistic functions $\beta _v = v/c \simeq 1$ and $\gamma = E_{\mathrm {beam}}/m_p$, $E_{\mathrm {beam}}$ is the beam energy and $m_p$ is the mass of the proton. The focusing of the beams is characterised by the $\beta$-function, and especially its minimum value $\beta ^*$. The longitudinal size of the luminous region is determined by the bunch length and by the angle $\phi$ (full crossing angle) at which the two beams are brought into collision. In the following discussion it is assumed that the emittances and $\beta$-functions in the horizontal and vertical direction are the same for each of the two beams. These assumptions lead to a circular transverse beam profile, as has been observed to be approximately the case at the LHC.

The particle densities in proton bunches can be described by three-dimensional Gaussian distributions with transverse and longitudinal sizes given by $\sigma _{x} =\sigma _{y} = \sqrt{\varepsilon _{} \, \beta _{}}$ and $\sigma _{z} = c \, T_z / 4$ respectively, where $T_z$ is the “four $\sigma$ bunch length” (in ns) customarily quoted for the LHC. Because the ratio $\sigma _{z}/\beta ^*$ was small during Run 1, the quadratic form of the $\beta$-function around the interaction region had a negligible effect over the length of the luminous region and the transverse beam size along the beam axis remained constant. As a result the luminous region is described well by a three-dimensional Gaussian distribution. With the assumption of pair-wise equal bunch sizes mentioned above, the transverse size $\sigma _{x{\mathcal L}}$ (and equivalently $\sigma _{y{\mathcal L}}$) of the luminous region is given by $\sigma _{x{\mathcal L}} = \sigma _{x} / \sqrt{2}$. For a crossing angle in the vertical plane as is the case for ATLAS, and assuming equal longitudinal bunch sizes $\sigma _{z}$ in both beams, the longitudinal size of the luminous region is given by:

3 $$\begin{aligned} \sigma _{z{\mathcal L}} = \frac{c \, T_z / 4}{\sqrt{2} } \frac{1}{\sqrt{1 \, + \, \left( \frac{\sigma _{z}}{\sigma _{y}}\frac{\phi }{2}\right) ^2}}. \end{aligned}$$

A summary of typical LHC parameters for *pp* collisions at $\sqrt{s} = 7\,\mathrm {TeV}$ in 2011 and at $\sqrt{s} = 8\,\mathrm {TeV}$ in 2012 is shown in Table 1 together with the resulting expected sizes of the luminous region. The measured sizes of the luminous region are discussed in Sect. 4.4 and Table 3.

**Table 1:** Summary of LHC parameters for typical *pp* collision fills and corresponding expected sizes of the luminous region. Emittance and bunch length values (and the corresponding beam-spot sizes) refer to values expected at the start of a fill. The two values given for expected transverse and longitudinal beam-spot size in 2011 correspond to the two $\beta ^*$ settings of 1.5 and 1.0 m. Measured average beam-spot parameters are presented in Table 3 (Sect. 4.4)

Year	2011	2012
Beam energy (TeV)	3.5	4.0
$\beta ^*$ (m)	1.5, 1.0	0.6
Normalised emittance $\varepsilon _{\text {N}}$ ($\upmu$m rad)	2.5	2.5
Full crossing angle $\phi$ ($\upmu$rad)	240	290
$4\sigma$ bunch length $T_z$ (ns)	1.20	1.25
Bunch length $\sigma _{z}$ (mm)	90	94
Expected transverse beam-spot size $\sigma _{x{\mathcal L}}$, $\sigma _{y{\mathcal L}}$ ($\upmu$m)	22, 18	13
Expected longitudinal beam-spot size $\sigma _{z{\mathcal L}}$ (mm)	60, 59	54

## Data and Monte Carlo samples

This paper uses *pp* collision data with $\sqrt{s} = 8$ TeV recorded during the LHC Run 1 period. Data were collected using the minimum-bias triggers described in Sect. 2. The data-taking conditions of the corresponding data samples are summarised in Table 2.

**Table 2:** The data-taking conditions of the *pp* collision data samples used in this paper

Pile-up conditions	$\mu$ range	Date
Low $\mu$	0–1	April 2012
High $\mu$	55–72	July 2012
Run 1 data range	7–40	2012

**Fig. 2:**
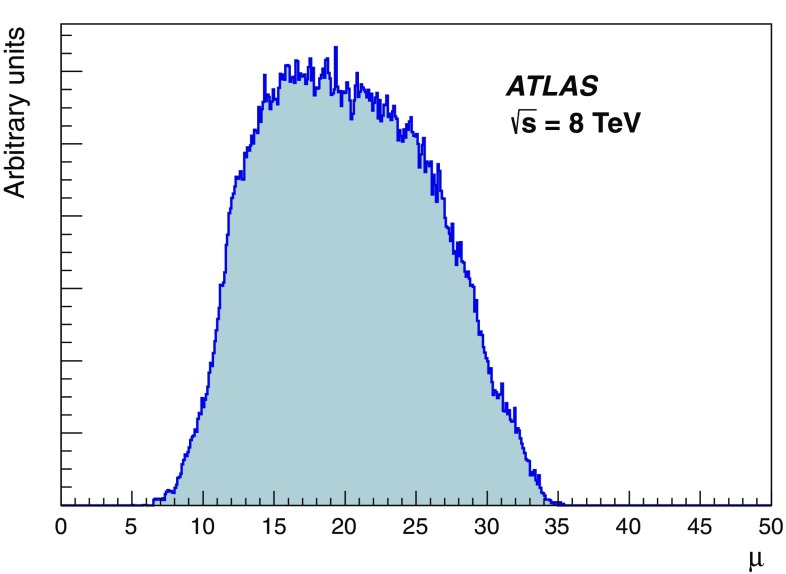
The average number of interactions per proton bunch crossing, $\mu$, during 8 TeV data-taking in Run 1, weighted by the luminosity

The studies presented here aim to cover the full range of Run 1 $\mu$ values and use both a special high-$\mu$ data sample as well as a range of lower-$\mu$ data. The distribution of the average number of interactions per *pp* bunch crossing in Run 1 is shown in Fig. 2.

This does not include the special high and low $\mu$ runs listed in Table 2. Most data taken in Run 1 had pile-up near $\mu = 20$. The low pile-up dataset was taken at average $\mu$ around 0.01, while the special high pile-up run featured peak collision multiplicities up to $\mu =72$.

The results presented in this paper use MC simulation of hard-scatter interactions and soft inelastic *pp* collisions. The collection of soft inelastic interactions is referred to here as the minimum-bias sample. These are events that would have been collected with the minimum-bias trigger, described in Sect. 2.2, and they represent an average beam crossing, without selection of a specific hard-scatter interaction.

Minimum-bias samples were simulated with the PYTHIA8 MC generator, with the A2 set of tuned parameters [10] and the MSTW2008LO parton density function set [11]. The PYTHIA8 model for soft QCD uses a phenomenological adaptation of $2\rightarrow 2$ parton scattering to describe low transverse momentum processes. Samples were generated for non-diffractive, single-diffractive, and double-diffractive interactions. These contributions were combined according to the PYTHIA8 generator cross sections.

To study the collective effects of multiple primary vertices reconstructed in one beam crossing, MC simulation with no hard-scattering process but only pile-up was created for $\mu$ up to 72. These samples mimic randomly triggered events, and were also generated with PYTHIA8 using the A2 tune. A special configuration was used to match 2012 data-taking conditions, including the beam spot with *z*-direction size equal to the average observed in data.

Hard-scatter interactions were simulated with POWHEG [12] interfaced to PYTHIA8 for the $Z\rightarrow \mu \mu$ and $H\rightarrow \gamma \gamma$ processes, and MC@NLO [13], HERWIG [14] and Jimmy [15] for top-quark pair production ($t\bar{t}$). The CT10 parameterisation [16] of the parton density functions was used. The top-quark pairs were generated with a lepton filter, requiring a lepton in the final state. The hard-scatter interaction samples were generated for a range of pile-up between $\mu =0$ and 38. The overlaid pile-up collisions were simulated with the soft QCD processes of PYTHIA8 in the manner of the minimum-bias simulation described above.

All generated events are processed with the ATLAS detector simulation framework [17], using the GEANT 4 [18] toolkit. After full detector simulation, the MC events are reconstructed and analysed in the same manner as data.

When comparing data with simulation in the presence of pile-up interactions, the average number of collisions per bunch crossing in simulation is re-weighted to match that measured in data. In order to obtain the same visible cross section for *pp* interactions for the simulation and data, a $\mu$-rescaling is also applied before the re-weighting. The rescaling factor is calculated by comparing the ratio of the visible cross section to the total inelastic cross section, $\epsilon _{\xi } = \sigma ^{\text {v}is}_{\text {i}nel} / \sigma _{\text {i}nel}$, for data with that for simulation. The value of $\epsilon _{\xi }^{\text {d}ata}$ is computed from independent measurements of these cross sections in data [19, 20]. The value of $\epsilon _{\xi }^{\text {M}C}$ is computed from events simulated with the PYTHIA8 MC generator with the A2 tune. The final scale factor is corrected to match the visible cross section within the ATLAS inner detector acceptance, resulting in $\epsilon ^{\text {M}C}_{\xi }/ \epsilon ^{\text {d}ata}_{\xi }=1.11$. The uncertainty in this scale factor is 5%. It is calculated from the quadrature sum of the uncertainties in the cross-section measurements, 3.5 and 2.6% from Refs. [19, 20] respectively, and a 2% uncertainty in the extrapolation from 7 to 8 TeV and to the inner detector acceptance.

## Primary vertex reconstruction

This section describes the method for reconstructing primary vertices. The input to the vertex reconstruction is a collection of reconstructed tracks. A brief summary of the main steps of track reconstruction is presented in Sect. 4.1. The vertex reconstruction is presented in Sect. 4.2. This is followed by a description of how primary vertices are used to reconstruct the shape of the luminous region, or beam spot, in Sect. 4.3, and a description of the stability of the beam spot in Sect. 4.4.

### Track reconstruction

The reconstruction of charged-particle trajectories in the inner detector is based on fitting a trajectory model to a set of measurements. The reconstructed charged-particle trajectories are hereafter referred to as tracks. The general structure and performance of ATLAS track reconstruction is described in detail in Refs. [21, 22] and a brief overview is given below.

Track seeds consist of three measurements in different layers of the pixel detector and SCT. Tracks are propagated out from the seed towards the TRT (“inside-out”) using a combinatorial Kalman filter [22], and additional silicon hits are added to the seed. An ambiguity solving procedure is applied to remove track candidates with incorrectly assigned hits. The candidate tracks are scored in a reward–penalty schema with respect to one another. To favour fully reconstructed tracks over short track segments, each additional measurement associated with a track leads to a better score value. The measurements from different sub-detectors are weighted differently, preferring the precision measurements (e.g. pixel clusters) and downgrading measurements from less precise detector parts. To provide a realistic description of detector acceptance and efficiency, the concept of a hole on a track is introduced. A hole represents a measurement on a detector surface that is expected, given the trajectory predictions, but not observed (holes are not considered on the first and last surfaces in the measurement). The presence of holes reduces the overall track score. The $\chi ^{2}$ of the track fit is also used to penalise poor-quality candidates. Finally, the logarithm of the track transverse momentum $\ln ({p_{\text {T}}})$ is considered as a criterion to promote energetic tracks and to suppress the larger number of tracks formed from incorrect combinations of clusters, which tend to have low measured $p_{\text {T}}$. After the reconstruction of tracks in the pixel and the SCT detectors, the successful candidates are extrapolated into the TRT volume and combined with measurements there.

During data-taking at $\sqrt{s}=8$ TeV, the input to the vertex reconstruction algorithms consisted of charged-particle tracks selected according to the following criteria:

- $p_{\text {T}} >400$ MeV; $|d_{0}|<4$ mm; $\sigma (d_{0})<5$ mm; $\sigma (z_{0})<10$ mm;
- At least four hits in the SCT detector;
- At least nine silicon (SCT or pixel) hits;
- No pixel holes.

Here the symbols $d_{0}$ and $z_{0}$ denote the transverse and longitudinal impact parameters of tracks with respect to the centre of the luminous region, and $\sigma (d_{0})$ and $\sigma (z_{0})$ denote the corresponding uncertainties [21]. The impact parameter requirements are applied to reduce contamination from tracks originating from secondary interactions. The above requirements are tighter than the standard ATLAS track selection criteria in order to maintain a low rate of fake tracks (tracks mistakenly reconstructed from a random combination of hits) at Run 1 pile-up levels (up to $\mu = 40$). The track reconstruction efficiency under this selection is between 75 and 85% for central rapidities ($|\eta | < 1.5$) and track $p_{\text {T}}$ above 500 MeV; the efficiency falls to about 60% at higher rapidities or about 65% for tracks with $p_{\text {T}}$ between 400 and 500 MeV.

### Primary vertex finding and fitting

The procedure of primary vertex reconstruction is divided into two stages: vertex finding and vertex fitting [23]. The former stage generally denotes the pattern recognition process: the association of reconstructed tracks to vertex candidates. The vertex fitting stage deals with reconstruction of the actual vertex position and its covariance matrix. The strategy is explained in detail in this section, and can be briefly outlined in these steps:

- A set of tracks satisfying the track selection criteria is defined.
- A seed position for the first vertex is selected.
- The tracks and the seed are used to estimate the best vertex position with a fit. The fit is an iterative procedure, and in each iteration less compatible tracks are down-weighted and the vertex position is recomputed.
- After the vertex position is determined, tracks that are incompatible with the vertex are removed from it and allowed to be used in the determination of another vertex.
- The procedure is repeated with the remaining tracks in the event.

Each of these steps (except the track selection described in the previous section) is expanded on below.

**Fig. 3:**
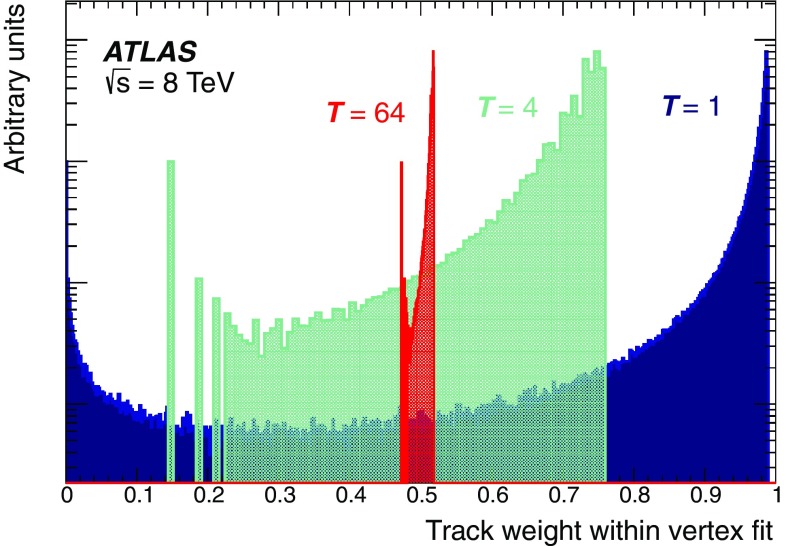
Histogram showing the weights applied to tracks in the vertex reconstruction fit. The fitting algorithm iterates through progressively smaller values of the temperature *T*, effectively down-weighting outlying tracks in the vertex fit. The vertical axis is on a logarithmic scale

1. The seed position of the vertex fit is based on the beam spot in the transverse plane. The *x*- and *y*-coordinates of the starting point are taken from the centre of the beam spot, reconstructed as discussed in Sect. 4.3. The *z*-coordinate of the starting point is calculated as the mode of the *z*-coordinates of tracks at their respective points of closest approach to the reconstructed centre of the beam spot. The mode is calculated using the Half-Sample Mode algorithm [24].
2. After the seed has been determined, the iterative primary vertex finding procedure begins. The vertex position is determined using an adaptive vertex fitting algorithm with an annealing procedure [25]. Using the seed position as the starting point and parameters of reconstructed tracks as input measurements, the algorithm performs an iterative $\chi ^2$ minimisation, finding the optimal vertex position. Each input track is assigned a weight, reflecting its compatibility with the vertex estimate. The vertex position is recalculated using the weighted tracks, and then the procedure is repeated, recalculating track weights with respect to the new vertex position. The individual track weights are calculated according to the following equation:
  4 $$\begin{aligned} \omega ( \hat{\chi }^2 ) = \frac{1}{1 + \exp \left( \frac{\hat{\chi }^{2} - \chi ^{2}_{\text {c}utoff}}{2T}\right) }. \end{aligned}$$
  Here $\hat{\chi }^{2}$ is the $\chi ^2$ value calculated in three dimensions between the last estimated vertex position and the respective point of the closest approach of the track. Tracks with lower weights are less compatible with the vertex and will have less influence on the position calculation. The constant $\chi ^{2}_{\text {c}utoff}$ defines the threshold where the weight of an individual track becomes equal to 0.5. Tracks with low weights are not removed, but will have less impact on the calculated vertex position. The value of $\chi ^{2}_{\text {c}utoff}$ is set to nine, which corresponds to about three standard deviations. The temperature *T* controls the smoothness of the weighting procedure. For low values of *T*, $\omega ( \hat{\chi }^2 )$ approaches a step function, and for large values of *T* the function flattens, progressively losing its $\chi ^2$ dependence. To avoid convergence in local minima, the weighting procedure is applied progressively by decreasing the temperature *T* during the fit iterations. The temperature is lowered from some high starting value in a pre-defined sequence of steps that converges at $T=1$. A typical distribution of track weights is shown in Fig. 3. It widens as *T* decreases, reaching an optimal separation of track outliers for $T = 1$.
3. After the last iteration, the final weight of each track used in the vertex fit is evaluated. Tracks found incompatible with the vertex by more than seven standard deviations are removed from the vertex candidate and returned to the pool of unused tracks. This loose requirement is intended to reduce the number of single *pp* interactions which are reconstructed as two distinct primary vertices due to the presence of track outliers, while maintaining a high efficiency.
4. After the vertex candidate is created, the rejected tracks are considered as input for a new vertex finding iteration. The procedure described above is then repeated starting from step 1, calculating the new starting position from remaining tracks, until no unassociated tracks are left in the event or no additional vertex can be found in the remaining set of tracks.

**Fig. 4:**
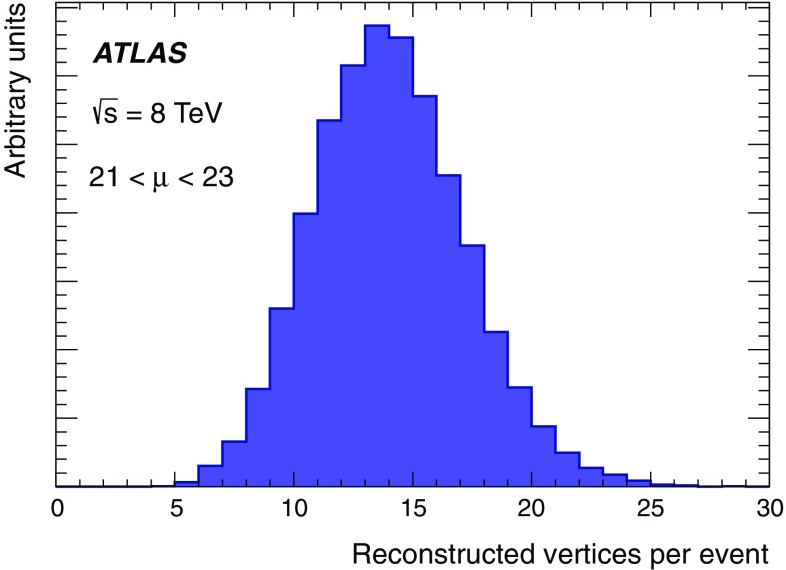
Distribution of the number of reconstructed vertices per event in a sample of $\sqrt{s}= 8$ TeV minimum-bias data for the pile-up range $21< \mu <23$

All vertices with at least two associated tracks are retained as valid primary vertex candidates. The output of the vertex reconstruction algorithm is a set of three dimensional vertex positions and their covariance matrices. Figure 4 shows a typical distribution for the number of reconstructed vertices per event in Run 1 for minimum-bias data collected in the pile-up range $21< \mu <23$.

**Fig. 5:**
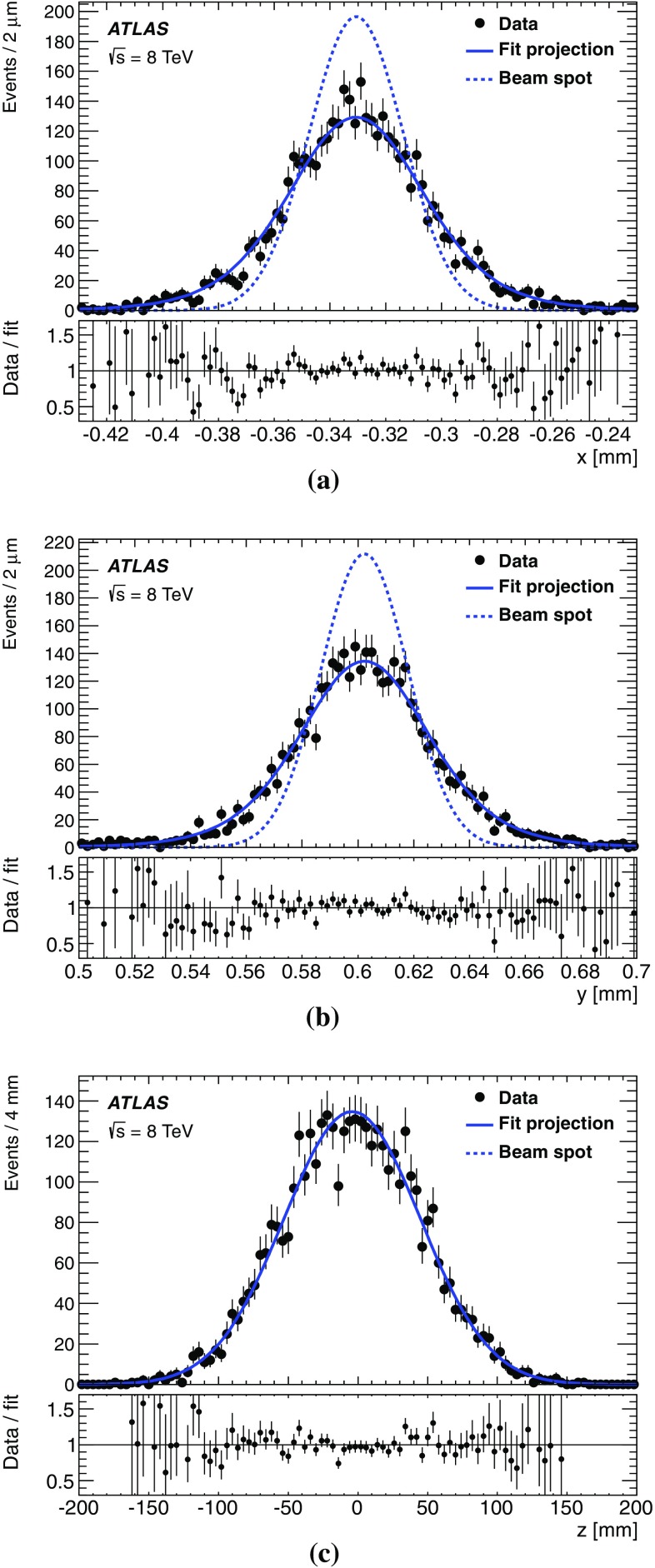
Distribution in **a** *x*, **b** *y* and **c** *z* of the reconstructed primary vertices used for a typical single beam-spot fit, projection of the 3D Gaussian beam-spot fit result, and fitted beam spot. The fit projection and beam spot curves are identical in **c**

The reconstructed position and width of the beam spot can be used as an additional measurement during the primary vertex fit. It is taken as a three-dimensional Gaussian measurement centred around the beam-spot centre and with the beam-spot size as the width. Tracks outside the beam spot have low compatibility with the vertex fit and are thus removed in the iterative fitting procedure. This procedure is hereafter referred to as the beam-spot constraint. Figure 5 shows typical distributions of the *x*, *y*, and *z* coordinates of primary vertices without the beam-spot constraint.

The transverse position resolution of vertices reconstructed from a small number of tracks may exceed $100\;\upmu {\text {m}}$. For these vertices the application of the beam-spot constraint significantly improves their transverse position resolution. In the *z*-direction, the length of the luminous region has no significant impact on the resolution of primary vertices. The longitudinal resolution of primary vertices is determined by the intrinsic resolution of the primary tracks. However, knowledge of the longitudinal beam-spot size still helps to remove far outlying tracks.

### Beam-spot reconstruction

The beam-spot reconstruction is based on an unbinned maximum-likelihood fit to the spatial distribution of primary vertices collected from many events. These primary vertices are reconstructed without beam-spot constraint from a representative subset of the data called the express stream during the detector calibration performed approximately every ten minutes. In each event only the primary vertex with the highest sum of squares of transverse momenta of contributing tracks, denoted hereafter as $\sum {p_{\text {T}} ^2}$, is considered. In order to be used in the beam-spot fit, this vertex must include at least five tracks and must have a probability of the $\chi ^2$ of the vertex fit greater than 0.1%. The requirement of at least five tracks ensures that most vertices have a transverse vertex resolution better than $50\,\upmu$m with a most probable value of about $15~\upmu$m that is comparable to the transverse beam-spot size. At least 100 selected vertices are required to perform a beam-spot fit, and in a typical fit several thousand vertices collected over a time period of about ten minutes are available. The fit extracts the centroid position ($\overline{x}_{\mathcal {L}}$, $\overline{y}_{\mathcal {L}}$, $\overline{z}_{\mathcal {L}}$) of the beam spot (luminous centroid), the tilt angles $\overline{x}_{\mathcal {L}} '$ and $\overline{y}_{\mathcal {L}} '$ in the *x*–*z* and *y*–*z* planes respectively, and the luminous sizes ($\sigma _{x{\mathcal L}}$, $\sigma _{y{\mathcal L}}$, $\sigma _{z{\mathcal L}}$), which are the measured sizes of the luminous region with the vertex resolution deconvoluted from the measurements.

In the transverse plane the width of the distribution of primary vertices is the convolution of the vertex resolution with the width of the luminous region. This is modelled by the transverse covariance matrix

5 $$\begin{aligned} V_{i} = V^{\text {B}} + k^2 \; V^\mathrm {V}_{i} \; , \end{aligned}$$

where $V^\mathrm {B}$ describes the transverse beam-spot size and allows for a rotation of the luminous-region ellipsoid in the transverse plane in case of non-circular beams. The transverse vertex resolution $V^\mathrm {V}_{i}$ estimated by the vertex fit for each primary vertex *i* is scaled by a parameter *k* determined by the beam-spot fit in order to account for any differences between fitted and expected vertex resolutions. The parameter *k* is expected to be close to unity as long as the vertex fitter provides good estimates of the vertex position uncertainty, the contamination from secondary vertices among the primary vertex candidates used in the beam-spot fit is small, and the Gaussian fit model provides an adequate description of the beam-spot shape. During 2012, the average value of *k* was 1.16. No vertex resolution correction and no error scaling is applied in the longitudinal direction because the longitudinal beam-spot size of about 50 mm is much larger than the typical *z* resolution of $35~\upmu$m for the vertices selected for the beam-spot fit.

The beam-spot fit assumes a Gaussian shape in *x*, *y* and *z* and the corresponding probability density function (PDF) is maximised using the Minuit [26] minimisation package after an iterative procedure removes a small number of outliers incompatible with the fit. The effect of this outlier removal on the fitted beam-spot parameters is negligible but brings the error scaling factor *k* closer to 1.

As an example of the beam-spot fit, Fig. 5 shows the distribution of primary vertices selected as input to the beam-spot fit (before outlier removal), together with the projection of the fit result. The fitted beam spot, i.e. the distribution of primary vertices after unfolding of the vertex position resolution, is also shown. The impact of the vertex position resolution is clearly seen in the transverse direction, whereas in the longitudinal (*z*) direction the vertex resolution is negligible compared to the beam spot and therefore fitted beam spot and fit projection are identical.

### Beam-spot stability

**Fig. 6:**
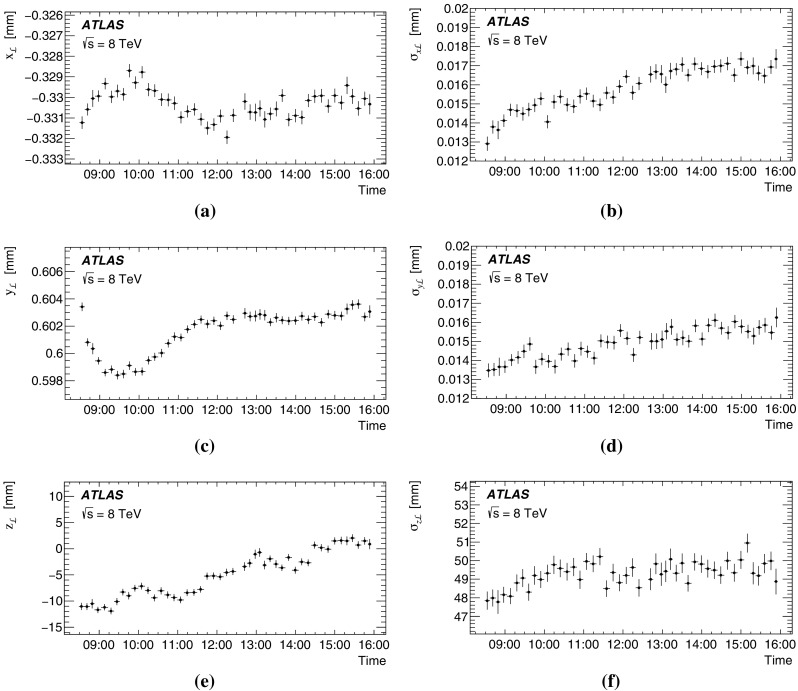
Position (**a** ${{x}_{\mathcal {L}}}$, **c** ${{y}_{\mathcal {L}}}$, **e** ${{z}_{\mathcal {L}}}$) and size (**b** $\sigma _{x{\mathcal L}}$, **d** $\sigma _{y{\mathcal L}}$, **f** $\sigma _{z{\mathcal L}}$) of the luminous region in ATLAS during a typical fill at $\sqrt{s} = 8$ TeV. The transverse sizes are corrected for the transverse vertex resolution

The evolution of the beam-spot position and size as a function of time during a typical LHC fill is shown in Fig. 6. The coordinates of the beam-spot position are given with respect to the ATLAS coordinate system. The precise origin location and the orientation of the ATLAS coordinate system is defined through the detector alignment procedure. The origin was chosen to be at the nominal interaction point with a *z*-axis along the beam direction, ensuring that the coordinates of the beam-spot centroid position are close to zero. In the early Run 1 data, a tilt angle of $\overline{x}_{\mathcal {L}}' \approx 500\, \upmu$rad was observed. In 2011 the ATLAS coordinate system was rotated in order to align the coordinate system more precisely with the beam line.

The downward movement of the beam-spot position during the first 40 min of the run followed by a gradual rise as seen in Fig. 6c is typical and is attributed to movement of the pixel detector after powering up from standby. The increase in transverse size during the fill (Fig. 6b, d) is expected from the transverse-emittance growth of the beams. The magnitude of the changes in longitudinal beam-spot position (Fig. 6e) is typical and is understood to be due to relative RF phase drift. The increase in longitudinal size (Fig. 6f) reflects bunch lengthening in the beams during the fill. The tilt angles $\overline{x}_{\mathcal {L}}'$ and $\overline{y}_{\mathcal {L}}'$ (not shown in Fig. 6) were stable at the level of about $10\, \upmu$rad.

**Fig. 7:**
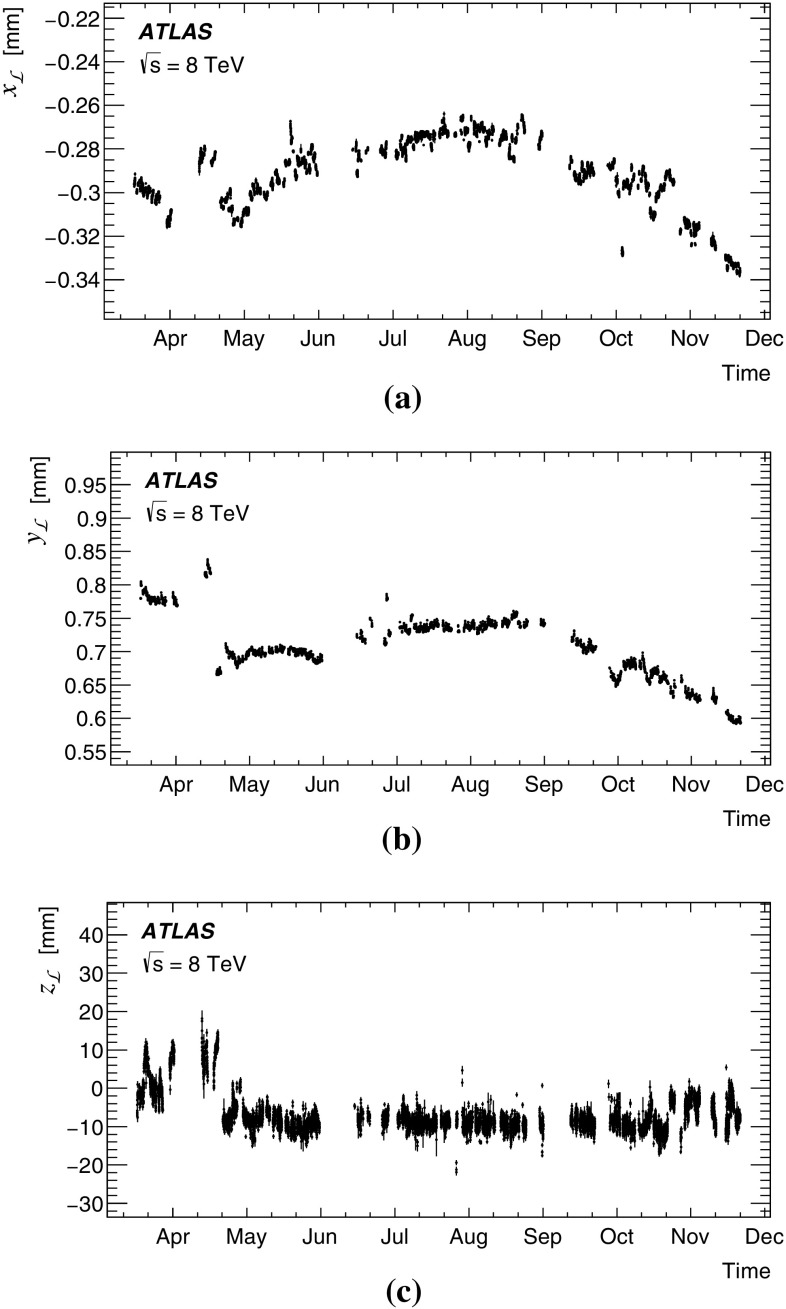
Position of the luminous region in ATLAS over the course of *pp* running in 2012 at $\sqrt{s}=8$ TeV. The data points are the result of a maximum likelihood fit to the spatial distribution of primary vertices collected over ten minutes. Errors are statistical only

The long-term evolution of the beam-spot position during 2012 is shown in Fig. 7. The large vertical movement at the beginning of May visible in Fig. 7b was associated with movement of the ID.

Apart from variations in each fill due to transverse-emittance growth and bunch lengthening, both the transverse and longitudinal beam-spot sizes remained unchanged during 2012.

Table 3 summarises the beam-spot position and size in 2010, 2011 and 2012 for *pp* collision data.

**Table 3:** Average beam-spot position and size for *pp* collision data in 2010, 2011 and 2012 for different $\beta ^*$ settings. The errors given in the table are the RMS spread of the parameters during the corresponding time period

Year	$\beta ^*$ (m)	$\overline{x}_{\mathcal {L}}$ (mm)	$\overline{y}_{\mathcal {L}}$ (mm)	$\overline{z}_{\mathcal {L}}$ (mm)	$\sigma _{x{\mathcal L}}$ ($\upmu$m)	$\sigma _{y{\mathcal L}}$ ($\upmu$m)	$\sigma _{z{\mathcal L}}$ (mm)
2010	11	$-0.347\pm 0.015$	$0.611 \pm 0.018$	$\phantom {-}0.9 \pm 3.5$	$49 \pm 8$	$60 \pm 12$	$29 \pm 3$
2010	2	$-0.364\pm 0.031$	$0.647 \pm 0.009$	$-1.2 \pm 2.2$	$30 \pm 5$	$39 \pm 12$	$36 \pm 3$
2010	3.5	$\phantom {-}0.081\pm 0.033$	$1.099 \pm 0.029$	$-3.0\pm 4.6$	$41 \pm 4$	$44 \pm 6$	$63 \pm 3$
2011	1.5	$-0.050\pm 0.018$	$1.059 \pm 0.051$	$-6.2 \pm 3.8$	$26 \pm 2$	$24 \pm 2$	$57 \pm 3$
2011	1.0	$-0.052 \pm 0.009$	$1.067 \pm 0.013$	$-6.7 \pm 1.5$	$21 \pm 2$	$20 \pm 1$	$56 \pm 3$
2012	0.6	$-0.291 \pm 0.016$	$0.705 \pm 0.046$	$-7.3\pm 4.7$	$15 \pm 2$	$15 \pm 1$	$48 \pm 2$

Data from special runs is excluded. As expected, the average transverse beam-spot size scales approximately with $\sqrt{\beta ^*/E_{\mathrm {beam}}}$, but is also influenced by changes in the normalised emittance and by the amount of emittance growth during the fills. In 2010 and 2011 the centre-of-mass energy was 7 TeV. In 2012 it increased to 8 TeV. During this time the crossing angle $\phi$ was increased from zero at the start of 2010 to $290\,\upmu$rad in 2012.

The measured transverse size of the beam spot at the start of a run is in good agreement with the values expected from the LHC machine parameters at the start of a fill (Table 1). This can be seen in Fig. 6. The average transverse size in 2012 shown in Table 3 ($15\; \upmu {\text {m}}$) is larger than the expected size of $13~\upmu$m from Table 1 due to emittance growth during the run. Within the relatively large uncertainty expected for the $4\sigma$ bunch length $T_z$ due to instrumental and non-Gaussian effects, the longitudinal beam-spot size is in reasonable agreement with expectations from the LHC parameters shown in Table 1.

## Hard-scatter interaction vertices

This section describes how both the reconstruction and identification efficiencies of hard-scatter primary vertices are evaluated using simulation. The impact of pile-up tracks and vertices on the performance is also estimated. A classification scheme based on MC generator-level information, denoted hereafter as truth-level information, is used to describe the level of pile-up contamination in reconstructed vertices from hard-scatter processes.

### Monte Carlo truth matching and classification of vertices

To study the performance of primary vertex reconstruction using MC simulation, a truth-matching algorithm has been developed, based on the generator-level particles associated to tracks contributing to reconstructed vertices. The procedure first classifies each reconstructed track used in a vertex fit. The compatibility criteria for track truth-matching are based on the fraction of hits used to reconstruct the track in each sub-detector that were produced by the generated primary particle as discussed in Ref. [21]. Each reconstructed track is classified as one of the following:

- A track matched to a hard-scatter interaction.
- A track matched to a pile-up interaction.
- An unmatched track. Such a tracks are considered random combinations of detector hits falsely identified as charged particle trajectories. These are referred to as fake tracks.

Tracks are matched to their primary generating interaction, i.e. tracks from secondary interactions are traced back to a hard-scatter or pile-up interaction. Based on the above classification, reconstructed vertices can be categorised. For each vertex, the sum of the weights assigned to all contributing tracks is normalised to unity. The fractional weights of individual tracks in each vertex are calculated. Vertices can then be put into one of the following exclusive categories:

- *Matched vertex* Tracks identified as coming from the same generated interaction contribute at least 70% of the total weight of tracks fitted to the reconstructed vertex.
- *Merged vertex* No single generated interaction contributes more than 70% of track weight to the reconstructed vertex. Two or more generated interactions contribute to the reconstructed vertex.
- *Split vertex* The generated interaction with the largest contribution to the reconstructed vertex is also the largest contributor to one or more other reconstructed vertices. In this case, the reconstructed vertex with the highest fraction of track $\Sigma p_{\text {T}}^{2}$ is categorised as matched or merged and the vertex or vertices with lower $\Sigma p_{\text {T}}^{2}$ are categorised as split.
- *Fake vertex* Fake tracks contribute more weight to the reconstructed vertex than any generated interaction.

This classification schema allows detailed studies of vertex reconstruction in a pile-up environment. The effects of splitting and merging of primary vertices as well as the influence of these effects on the vertex reconstruction efficiency and primary vertex resolution can be studied. This schema also allows the reconstructed vertices to be associated either with the primary hard-scatter *pp* collision or with pile-up interactions.

When studying the hard-scatter *pp* collisions, the reconstructed events are classified based on the following mutually exclusive definitions:

- *Clean* The event contains one matched vertex corresponding to the hard-scatter interaction. The hard-scatter interaction does not contribute more than 50% of the accumulated track weight to any other vertex.
- *Low pile-up contamination* The event contains one and only one merged vertex where the hard-scatter interaction contributes more than 50% of the accumulated track weight.
- *High pile-up contamination* The event does not contain any vertex where the hard-scatter interaction contributes more than 50% of the accumulated track weight. It does however contain at least one merged vertex in which the hard-scatter interaction contributes between 1 and 50% of the accumulated track weight.
- *Split* The event contains at least two merged vertices in which the hard-scatter interaction contributes more than 50% of the accumulated track weight.
- *Inefficient* The event does not contain any vertex where the hard-scatter interaction contributes more than 1% of the accumulated track weight.

In the current analysis, all categories except “Inefficient” are considered as successful in reconstructing the hard-scatter primary vertex. All of these categories thus contribute to the calculation of total vertex reconstruction efficiency.

### Vertex reconstruction and selection efficiency for hard-scatter interactions

**Fig. 8:**
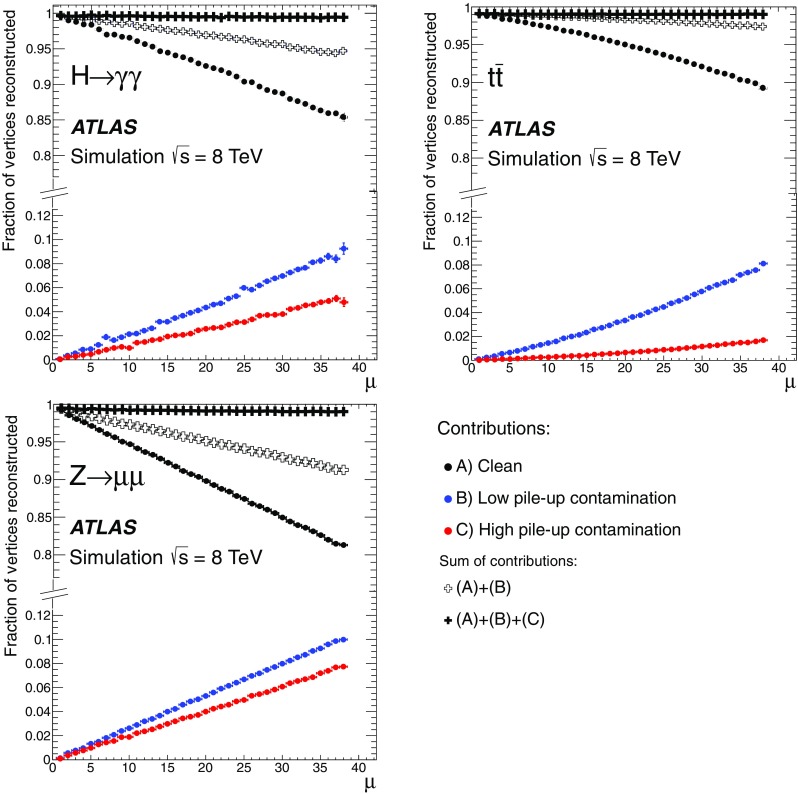
Contributions to the predicted primary vertex reconstruction efficiency as a function of the average number of interactions per bunch crossing, $\mu$. The mutually exclusive categories of events are defined in Sect. 5.1. The *black circles* show the contribution to the efficiency from events categorised as clean, and the *blue* and *red circles* show the contributions from events with low and high pile-up contamination respectively. The *open crosses* show the sum of the contributions from events that are clean and those with low pile-up contamination; the *filled crosses* show the sum of the contributions from all categories and represent the overall efficiency. The hard-scatter processes considered are Higgs-boson decay into $\gamma \gamma$, $t\bar{t}$ production with a lepton in the decay, and *Z*-boson decay into $\mu \mu$

The efficiency to reconstruct and also to correctly identify the hard-scatter primary vertex is used to quantify the impact of pile-up contamination. Assuming that the hard-scatter primary vertex produces reconstructed tracks, the efficiency of hard-scatter primary vertex reconstruction is predicted to be larger than 99%. This includes interactions with low or high pile-up contamination, and split event categories as defined in Sect. 5.1. The corresponding contributions to the reconstruction efficiencies as a function of simulated $\mu$ are shown in Fig. 8 for the processes $Z\rightarrow \mu \mu$, $H\rightarrow \gamma \gamma$ and $t\bar{t}\rightarrow l+X$ ($t\bar{t}$ decays that include a lepton).

The fraction of events with low and high pile-up contamination increases with growing $\mu$, while the fraction of clean events decreases with $\mu$. The fraction of events containing split vertices remains negligible for all $\mu$. For $\mu = 38$ the fraction of high pile-up contamination vertices is 8% for $Z\rightarrow \mu \mu$ events, 5% for $H\rightarrow \gamma \gamma$ events, and 2% for $t\bar{t}$ events.

The effect of pile-up contamination on the reconstruction efficiency for the hard-scatter primary vertex clearly depends on the nature of the physics process under study. The hard-scatter interactions corresponding to *Z*-boson production leave on average fewer charged particles within the detector acceptance than those corresponding to $t\bar{t}$ production. Hard-scatter vertices from *Z*-boson production can therefore be expected to be more affected by pile-up contamination than those from $t\bar{t}$ events. Indeed, Fig. 8 shows that the low and high pile-up contamination fractions are always higher for $Z\rightarrow \mu \mu$ than for $t\bar{t}$ events.

**Fig. 9:**
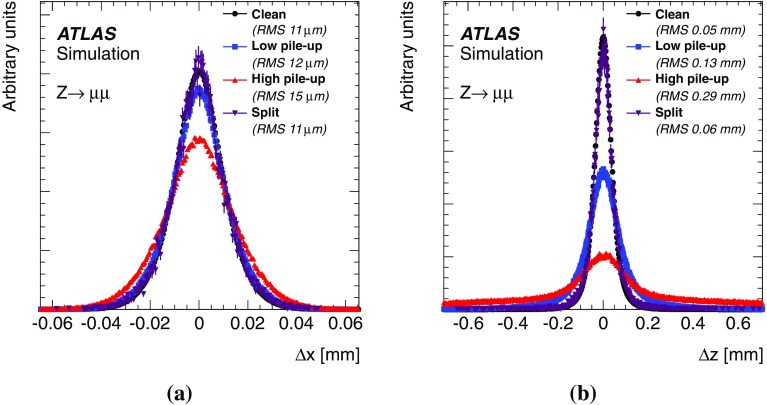
The residual distributions in **a** *x* and **b** *z* coordinates for reconstructed primary vertices in a sample of simulated $Z\rightarrow \mu \mu$ events for the four classes of events defined in Sect. 5.1. The distributions are normalised to the same area. The RMS values of these residuals are provided for each class

Pile-up tracks contaminating reconstructed hard-scatter vertices lead to a degradation of position resolution. Figure 9 shows the distribution of residuals of the primary vertex position in a $Z\rightarrow \mu \mu$ sample for different classes.

The residuals are calculated as the distance between the position of the hard-scatter primary vertex at generator level and its reconstructed position obtained from the primary vertex reconstruction as described in Sect. 4.2. Only the vertices matched according to the definition presented in Sect. 5.1 are taken into account. The results are obtained using the MC simulation including detector acceptance without further selection criteria. The categories of clean reconstruction, low and high pile-up contamination show progressively degrading resolution. This effect is visibly largest for the *z*-coordinate, because the transverse coordinates are constrained by the beam-spot width. The events categorised as containing split vertices do not suffer from a degraded resolution compared to the clean event category.

**Fig. 10:**
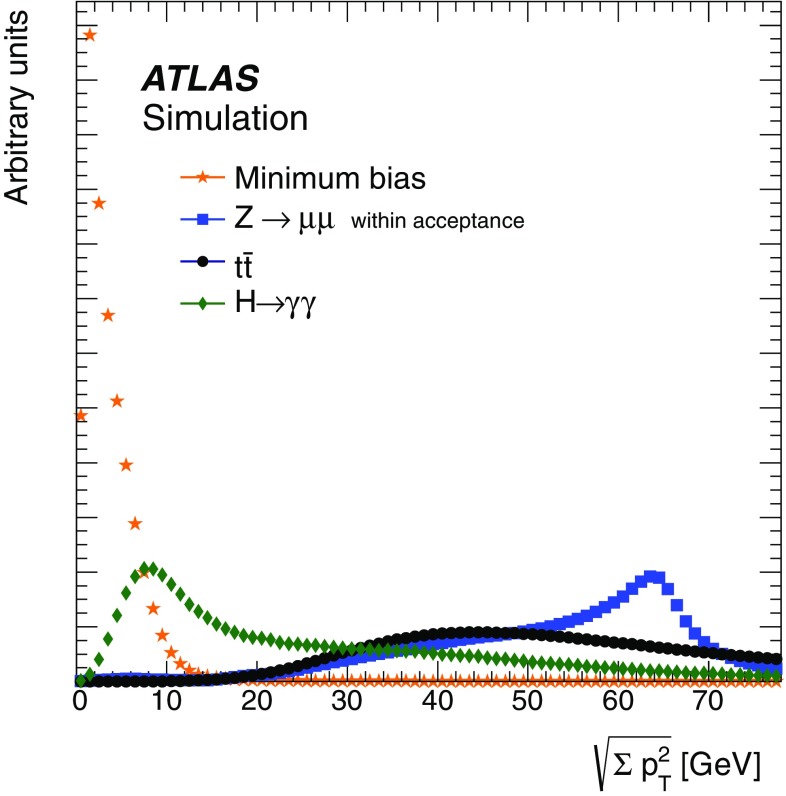
The distributions of the sum of the squared transverse momentum for tracks from primary vertices, shown for simulated hard-scatter processes and a minimum-bias sample. In the case of the $Z\rightarrow \mu \mu$ process, only events with at least two muons with $p_{\text {T}}>15$ GeV reconstructed within the ATLAS inner detector acceptance are shown. The $t\bar{t}$ process is filtered to select decays with leptons. The distributions are normalised to the same area

In addition to the degradation of the spatial resolution, the presence of significant pile-up makes it more difficult to correctly identify the hard-scatter primary vertex among the many pile-up vertices reconstructed in most bunch crossings. For most hard-scatter physics processes, it is effective to identify the hard-scatter primary vertex as the primary vertex with the highest sum of the squared transverse momenta of contributing tracks: $\sum p_{\text {T}} ^2$. This criterion is based on the assumption that the charged particles produced in hard-scatter interactions have on average a harder transverse momentum spectrum than those produced in pile-up collisions. The efficiency of the hard-scatter identification using this criterion depends on the kinematics of the hard-scatter process. Distributions of $\sqrt{\sum p_{\text {T}} ^2}$ of the tracks in various hard-scatter processes are shown in Fig. 10, including $H \rightarrow \gamma \gamma$, $Z\rightarrow \mu \mu$, and $t\bar{t}$ decays in which a filter has been applied to select decays with leptons. These are compared to a minimum-bias sample, which can be taken to have the same $\sqrt{\sum p_{\text {T}} ^2}$ distribution as pile-up.

In the case of $Z\rightarrow \mu \mu$ and $t\bar{t}$, there is significant transverse momentum carried by charged particles even in the case of inclusive samples. In contrast, in the case of $H \rightarrow \gamma \gamma$ events, most of the transverse momentum is carried by the photons from the Higgs boson decay. The remaining charged particles in the acceptance of the detector are produced in the underlying event and have a much softer $p_{\text {T}}$ spectrum. The efficiency to correctly select the hard-scatter vertex among many pile-up vertices by choosing the vertex with the highest $\sum p_{\text {T}} ^2$ is thus inferior for $H\rightarrow \gamma \gamma$ decays compared to most other hard-scatter processes. A more efficient method for choosing the primary vertex in the case of $H\rightarrow \gamma \gamma$ decay is described in Ref. [27].

**Fig. 11:**
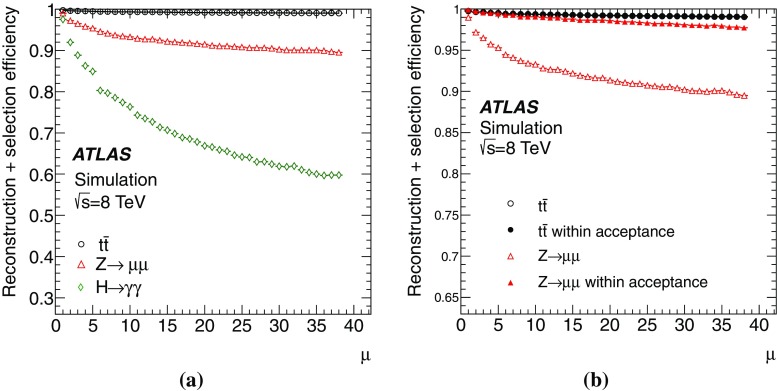
Efficiency to reconstruct and then select the hard-scatter primary vertex as a function of the average number of *pp* interactions per bunch crossing, $\mu$, for different physics processes: **a** all reconstructed events; **b** events with at least two muons with $p_{\text {T}}>15$ GeV reconstructed within the ATLAS inner detector acceptance. The points showing the $t\bar{t}$ efficiency with and without acceptance criteria overlap

For hard-scatter processes, the primary vertex selection efficiency is defined as the fraction of events in which the highest $\sum p_{\text {T}} ^2$ vertex is the vertex associated with the MC simulation hard scatter. The MC hard scatter is taken as the vertex with the highest weight of hard-scatter tracks, as described in Sect. 5.1. The efficiency to reconstruct and then select the hard-scatter primary vertex is shown as a function of $\mu$ in Fig. 11a for different physics processes.

The highest efficiency is achieved for $t\bar{t}$ events for all values of $\mu$. This observation is attributed to the high multiplicity of high transverse momentum tracks produced in top-quark decays. The selection efficiency for $Z\rightarrow \mu \mu$ events is greatly improved when additional criteria reflecting the kinematics of the physics process are imposed. Figure 11b shows the selection efficiencies after requiring at least two muons with $p_{\text {T}}>15$ GeV to be reconstructed within the ATLAS inner detector acceptance. The $t\bar{t}$ sample shows a selection efficiency above 99% with or without the muon acceptance requirement (the points are overlapping in the figure). A clear selection efficiency improvement for the $Z\rightarrow \mu \mu$ process is visible when muons are reconstructed in the acceptance, resulting in at most 2% of events with a wrongly selected hard-scattering primary vertex for $\mu$ of 38. These losses are primarily due to the small but non-zero probability that the $\sum p_{\text {T}} ^2$ of tracks from one of the inelastic interactions in the minimum-bias sample is larger than in the $Z\rightarrow \mu \mu$ interaction, as illustrated in Fig. 10. A more quantitative prediction of this loss is given in Sect. 8.

## Primary vertices in minimum-bias data

This section presents a study of single primary vertex reconstruction in soft interactions which are characteristic of the pile-up events superimposed on the hard-scatter event of interest. This study is based on a minimum-bias data sample with a single primary vertex reconstructed in each event and corresponding to an average number of interactions per bunch crossing $\mu = 0.01$. These data are compared to a simulation of inelastic interactions using the PYTHIA8 event generator.

The reconstruction efficiency for primary vertices produced in soft *pp* interactions varies depending on the nature of the soft interaction process. If the majority of final-state charged particles are produced outside the detector acceptance, the reconstruction of the corresponding primary vertex may be unsuccessful. The vertex reconstruction efficiency may be further reduced by the inefficient reconstruction of very low $p_{\text {T}}$ trajectories, characteristic of these soft interactions. Table 4 shows the efficiencies for reconstructing the primary vertex in events from a minimum-bias sample with only single interactions.

**Table 4:** Vertex reconstruction efficiencies, at various selection levels, for non-diffractive, single-diffractive, and double-diffractive interactions in PYTHIA8 minimum-bias simulation

	Non-diffractive (%)	Single-diffractive (%)	Double-diffractive (%)
Efficiency without any selection cuts	92.9	45.7	49.0
Efficiency requiring at least two charged particles with $p_{\text {T}}> 400$ MeV and $|\eta |< 2.5$	96.1	92.6	90.2
Efficiency requiring at least two charged particles reconstructed in the inner detector	99.6	99.5	99.3

These efficiencies are obtained from PYTHIA8 MC simulation separately for the three processes which produce minimum-bias triggers in the experiment, namely non-diffractive, single-diffractive, and double-diffractive interactions. Without selection cuts the reconstruction efficiency depends strongly on the process: increasing from 46% for single-diffractive to 93% for non-diffractive interactions. Taking into account the relative contributions of each process to inelastic interactions, the average efficiency is estimated to be about 80%. The difference in the efficiencies estimated for the different processes is primarily due to the different distributions of transverse momenta and pseudorapidities of charged particles produced in each process. In diffractive processes, the charged particles are mostly produced at large pseudorapidities, often outside the acceptance of the ATLAS tracking system. The very soft transverse momentum spectrum of these charged particles is an additional complication in their reconstruction. As shown in the second row of Table 4, basic geometrical and kinematic requirements on the generated particles remove most of the differences in efficiency among the non-diffractive, single- and double-diffractive processes. The overall vertex reconstruction efficiency increases to 95% in this case. The remaining differences in efficiencies are mostly due to the dependence of the track reconstruction efficiency on $\eta$ and $p_{\text {T}}$. The third row of Table 4 shows that the primary vertex reconstruction efficiency further increases to about 99% for all processes after requiring that at least two tracks are reconstructed within the inner detector, in addition to the requirements listed in the second row. The intrinsic efficiency of the ATLAS vertex reconstruction algorithm is thus expected to be very high if at least two charged particles are produced within the inner detector acceptance.

**Fig. 12:**
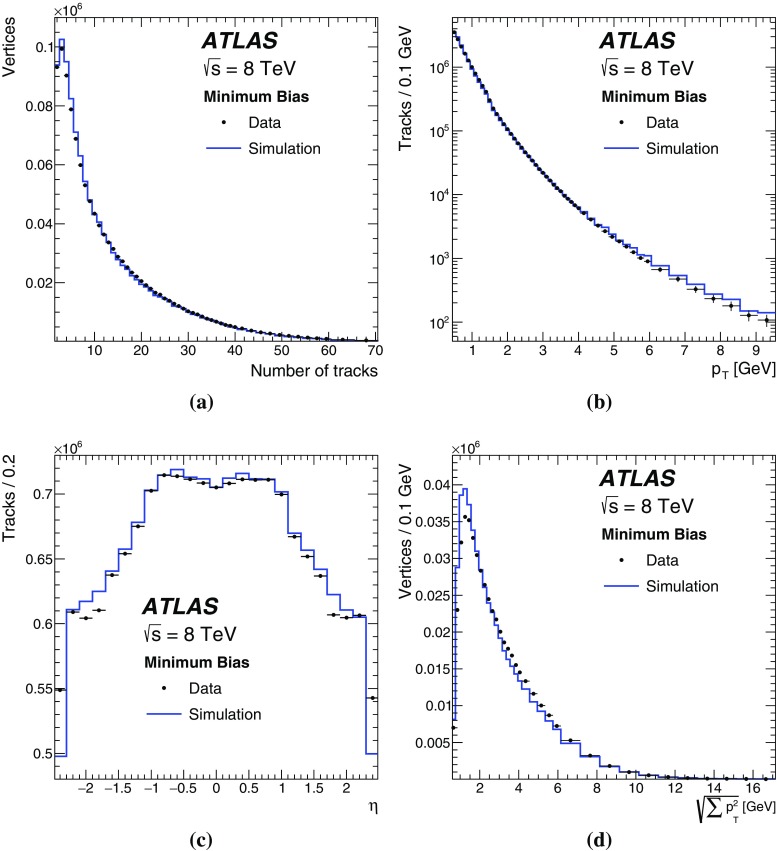
Distributions of **a** number of tracks per vertex, **b** track transverse momentum $p_{\text {T}}$, **c** track pseudorapidity $\eta$ and **d** $\sqrt{ \Sigma p_{\text {T}}^{2}}$ of the tracks associated with each vertex. Distributions are shown for tracks associated with primary vertices in low $\mu$ minimum-bias data and in simulation samples

Figure 12 compares the simulation to data for the distributions of the number of fitted tracks, the track $p_{\text {T}}$, track $\eta$, and $\sqrt{ \Sigma p_{\text {T}}^{2}}$ of tracks in primary vertices.

The figure illustrates how soft the pile-up interactions are: only 0.4% of the tracks belonging to a reconstructed primary vertex have $p_{\text {T}} > 4$ GeV and only 1.2% of the reconstructed vertices have a total $\sqrt{ \Sigma p_{\text {T}}^{2}}$ above 10 GeV. There are small discrepancies between simulation and data at very high values in the track $p_{\text {T}}$ spectrum and at high $\eta$. As described in Refs. [4, 10], these are due to deficiencies in the physics modelling of these distributions and not related to the primary vertex reconstruction algorithm. The dominant sources of systematic uncertainties relevant to the comparisons in Fig. 12 are the knowledge of the beam-spot size, the modelling of fake tracks, and the dependence of the track reconstruction efficiency on $p_{\text {T}}$, $\eta$ and $\mu$. These sources are not included in the error bars of the corresponding plots, but contribute to the observed discrepancies between data and simulation.

The position resolution of single vertices is estimated either from MC simulation or from data using the split-vertex method (SVM). In this method the *n* tracks associated to a primary vertex are ordered in descending order of their transverse momenta. The tracks are then split into two groups, one with even-ranking tracks and one with odd-ranking tracks, such that both groups have, on average, the same number of tracks, *n*/2. The vertex fit is applied independently to each group. The spatial separation between two resulting vertices gives a measurement of the intrinsic resolution for a vertex with *n*/2 tracks. The two split vertices must be reconstructed independently and therefore no beam-spot constraint is used during the fit.

**Fig. 13:**
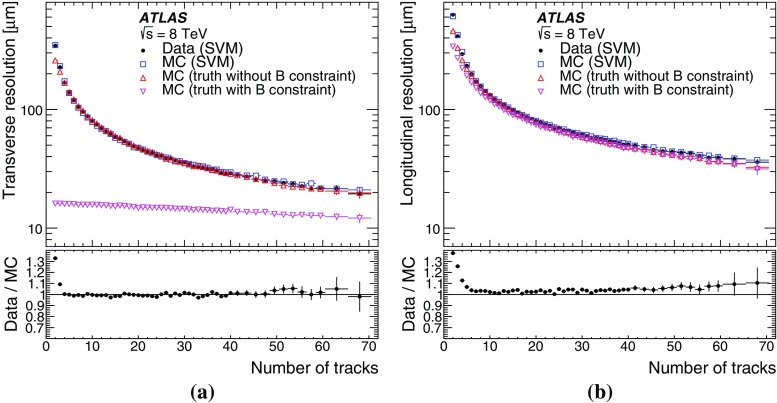
Resolution of the primary vertex position in **a** *x* and **b** *z* as function of the number of fitted tracks, estimated using the split-vertex method (SVM) for minimum-bias data (*black circles*) and MC simulation (*blue squares*). Also shown is the resolution obtained from the difference between the generator-level information and reconstructed primary vertex position in MC simulation (labeled “truth”), with and without the beam-spot constraint (*pink* and *red triangles* respectively). The *bottom panel* in each plot shows the ratio of the resolution found using the split-vertex method in data to that obtained using the MC generator-level information without the beam-spot constraint

Figure 13 shows the resolution in data calculated with the split-vertex method as a function of the number of tracks per vertex.

The split-vertex method is also used to calculate the resolution for the minimum-bias simulation sample. There is good agreement between the data and simulation distributions, showing that the reconstructed track parameters used in the vertex reconstruction are well modelled in the simulation. Figure 13 also shows the primary vertex resolution calculated as the difference between the true and reconstructed vertex position in the MC simulation. The good agreement between the split-vertex method and the resolution calculated with the MC generator-level information gives confidence that the split-vertex method provides a reliable measurement of the primary vertex resolution. At very low track multiplicity the result of the split-vertex method deviates slightly from the resolution obtained using the generator-level information. Here the resolution obtained from the generator-level information benefits from the perfect knowledge of vertex position decreasing the resolution spread, compared to the resolution obtained from the two reconstructed vertices in the split-vertex method. When the beam-spot constraint is included the resolution improves considerably in the transverse direction, staying below 20 $\upmu {\text {m}}$ for the full range of $\mu$ studied. The longitudinal resolution reaches 30 $\upmu$m at high track multiplicity. Figure 13 also shows the resolution calculated using MC generator-level information with and without beam-spot constraint.

## Performance in the high pile-up regime

In this section, the study of the primary vertex reconstruction performance at low $\mu$ is extended to the high pile-up regime. A dedicated data sample of minimum-bias events collected with values of $\mu$ between 55 and 72 was used to study the performance of the primary vertex reconstruction in the presence of multiple vertices. The simulation samples spanned values of $\mu$ from 0 to 22, typical of the standard 2012 data-taking conditions, and from 38 to 72 to emulate the high $\mu$ data sample.

**Fig. 14:**
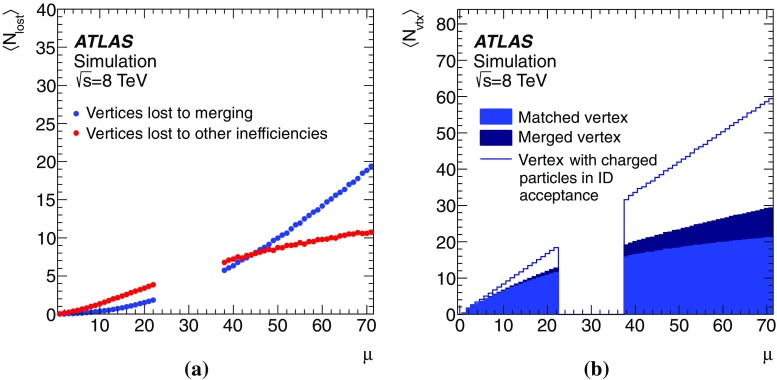
**a** Average number of generated primary vertices with at least two charged particles within the detector acceptance, that are not reconstructed due to merging (*blue*) and due to detector inefficiencies (*red*), as a function of the average number of interactions per bunch crossing, $\mu$. **b** Average number of reconstructed primary vertices of each truth-matching category compared to the total number of generated vertices with two particles within the detector acceptance, as a function of the average number of interactions per bunch crossing. The available MC simulation samples were generated with values of $\mu$ below 22 and above 38

The efficiency of primary vertex reconstruction decreases with increasing pile-up. In addition to the inefficiencies affecting single vertex reconstruction described in Sect. 6, effects related to the merging of adjacent primary vertices start to play a significant role as pile-up increases. Figure 14a shows the average number of vertices lost due to merging and to other effects, such as track reconstruction and detector acceptance.

Merging has a small effect on overall vertex reconstruction efficiency for $\mu$ values below 20, but it is a dominant effect for $\mu$ values above 40. Figure 14b shows the average number of expected reconstructed primary vertices as a function of $\mu$, for the two main classes of vertices defined in Sect. 5, matched vertices, consisting of tracks mostly coming from a single interaction, and merged vertices. For the highest values of $\mu$ around 70, where one expects about 60 primary vertices with at least two charged particles with $p_{\text {T}} > 400$ MeV within the detector acceptance, a total of 30 primary vertices are expected to be reconstructed on average, out of which about 10 are merged vertices. About 20 additional primary vertices are lost due to merging and about 10 due to other inefficiencies as shown in Fig. 14a. Vertices classified as “Fake” or “Split”, according to the definitions presented in Sect. 5.1, are not shown in Fig. 14b, since they represent a very small contribution of at most 2% of the total number of reconstructed vertices at $\mu = 70$.

**Fig. 15:**
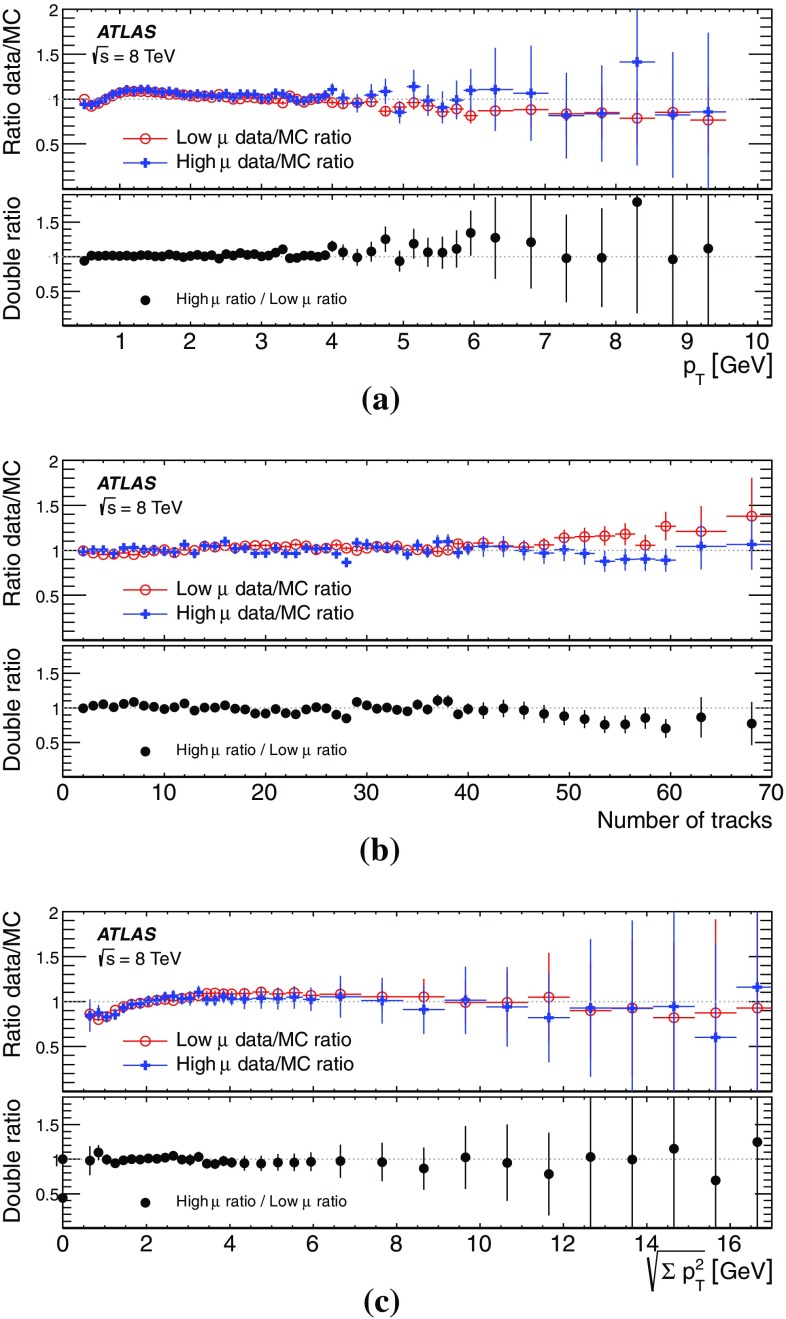
Ratios of data to MC simulation for observables relevant to the primary vertex reconstruction performance: **a** track transverse momentum $p_{\text {T}}$, **b** number of tracks per vertex, **c** $\sqrt{ \Sigma p_{\text {T}}^{2}}$ of the tracks in each vertex. *Error bars* represent only statistical uncertainties. The ratios are shown for low (0–1) and high (55–72) values of $\mu$. The *bottom panel* in each figure shows the double ratio of high to low $\mu$

The main observables relevant to the primary vertex reconstruction performance are in reasonable agreement between data and simulation with only small discrepancies attributed to the physics modelling of soft interactions (see Fig. 12). To quantify the agreement between data and simulation at high values of $\mu$, the same observables are studied and the ratios of data to simulation are compared between low and high values of $\mu$. This is shown in Fig. 15 for the track $p_{\text {T}}$, the number of tracks per primary vertex, and the $\sqrt{ \Sigma p_{\text {T}}^{2}}$ per primary vertex. The data to simulation ratios are overlaid for low and high $\mu$ samples in the upper panels. The lower panels show the double ratios of data to simulation between high and low values of $\mu$.

The double ratios agree with unity, showing that there is similar agreement between data and simulation at low and high $\mu$. In the case of track multiplicity, the agreement between data and simulation for high track multiplicities is somewhat better at high $\mu$ than at low $\mu$. This arises possibly because discrepancies in physics modelling are diluted by the contributions from merged vertices as $\mu$ increases.

## Efficiency of vertex reconstruction as a function of pile-up

An analytical model to predict the number of reconstructed vertices as a function of event multiplicity has been developed. This model is based on the measured primary vertex reconstruction efficiency and on the the probability of vertex merging.

### Modelling the number of reconstructed vertices

In the ideal case of perfect reconstruction efficiency, the number of reconstructed vertices would scale linearly with $\mu$. In reality there are a number of effects that cause the relation to be non-linear. As discussed in Sect. 7, one of the most important effects is vertex merging, when two or more vertices are merged and reconstructed as one vertex. Other effects include reconstruction inefficiencies, detector acceptance, and, at a small level for low track multiplicities, non-collision background. As already mentioned, the impact of fake and split vertices is negligible.

The average number of reconstructed vertices, $\langle n_{\text {V}ertices} \rangle$, can be parameterised as a function of $\mu$ as follows:

6 $$\begin{aligned} \langle n_{\text {V}ertices} \rangle = p_{0} + \epsilon \mu - F( \epsilon \mu , p_{\text {m}erge}), \end{aligned}$$

where $\epsilon$ is the efficiency of the vertex reconstruction algorithm before including vertex merging effects, and $p_{0}$ accounts for any small offset arising from non-collision background. Based on the results shown in Sects. 5, 6, and 7, the value of $\epsilon$ is considered to be independent of $\mu$. The quantity $\epsilon \mu$ represents the average number of vertices that would be reconstructed in the absence of any pile-up induced vertex merging effects. This quantity is referred to, hereafter, as the number of reconstructible vertices. In this study the parameter $\epsilon$ is obtained from a fit to the MC simulation. The function $F( \epsilon \mu , p_{\text {m}erge})$ represents the average number of vertices lost due to merging effects, taking into account the number of reconstructible vertices and the vertex merging probability, $p_{\text {m}erge}$. These effects are primarily responsible for the non-linear dependence of the number of reconstructed vertices as a function of $\mu$. The evaluation of this function is described in the next section.

The proposed model only describes the primary vertex reconstruction and does not account for pile-up effects in the reconstruction of tracks. The model assumes that the track reconstruction efficiency and the corresponding fake rate are constant for the studied range of pile-up values.

### Determination of correction for merging of primary vertices

**Fig. 16:**
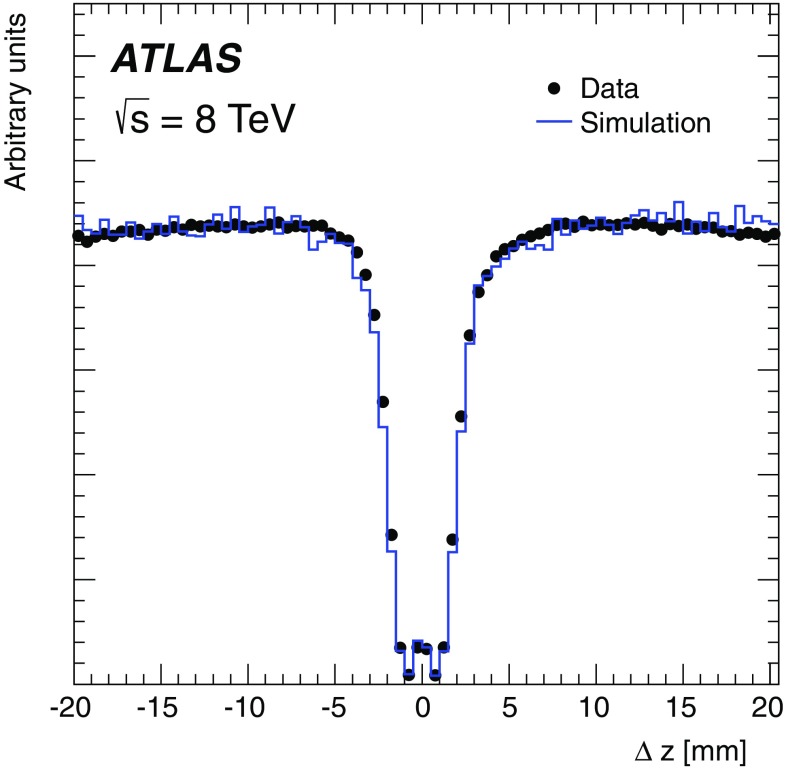
Distribution of the longitudinal separation between pairs of adjacent primary vertices in a typical Run 1 minimum-bias data sample and in MC simulation

The effects of vertex merging are studied using the longitudinal separation, $\Delta z$, between pairs of adjacent reconstructed primary vertices. The distribution of $\Delta z$ in a typical Run 1 minimum-bias data sample is shown in Fig. 16 together with the prediction from simulation.

At low values of $\Delta z$ close-by vertices can no longer be separated and are reconstructed as a single vertex. In Fig. 16, this effect is visible as a steep decrease of the number of reconstructed vertices at values of $\Delta z$ below a few mm. The small peak around $\Delta z = 0$ is due to the effect of splitting of primary vertices: in this case, close-by vertices are reconstructed with longitudinal separations well below the typical primary vertex resolution. The distribution of $\Delta z$ measured in a low pile-up data sample ($\mu$ below 10) is used to derive a two-vertex merging probability density function $p_{\text {m}erge}(\Delta z)$. This function can then be combined with a given beam-spot shape to derive an analytical relationship between the number of reconstructible vertices per event, $\epsilon \mu$, and the average number of reconstructed vertices, $\langle n_{\text {V}ertices}\rangle$. Using this approach, the effect of different beam-spot sizes on the merging probability can then also be evaluated.

The analytical function is derived as follows:

1. The $\Delta z$ distribution for pairs of adjacent vertices reconstructed in low pile-up data is fitted with a Gaussian function in a range where the merging of vertices is negligible: $|\Delta z| > 30$ mm. The Gaussian has an expected width of $\sqrt{2} \sigma _{z{\mathcal L}}$, where $\sigma _{z{\mathcal L}}$ is the longitudinal beam-spot RMS, assuming the beam spot has a Gaussian shape distribution along the *z*-axis.
2. A merging probability density function, $p_{\text {m}erge}(\Delta z)$, is constructed by taking the difference between the distribution of $\Delta z$ observed in data in the range $|\Delta z| < 30$ mm and the prediction obtained from the Gaussian fit, $f_{\text {e}xp}(\Delta z)$. This difference is then normalised to the prediction probability density function:
  7 $$\begin{aligned} p_{\text {m}erge}(\Delta z) = \frac{f_{\mathrm {exp}}(\Delta z) - f_{\mathrm {\text {o}bs}}(\Delta z)}{f_{\mathrm {\text {e}xp}}(\Delta z)}. \end{aligned}$$
  Here, $f_{\text {o}bs}(\Delta z)$ represents the observed probability density function of $\Delta z$ in the range $|\Delta z| < 30$ mm. An example of the observed distribution $f_{\text {o}bs}(\Delta z)$ is shown in Fig. 16. The $p_{\text {m}erge}(\Delta z)$ PDF is parameterised using a step function convolved with a Gaussian function with parameters fit to the observed distribution. The $p_{\text {m}erge}(\Delta z)$ PDF is derived in the low pile-up regime, where only the merging of adjacent pairs of vertices is assumed to be significant. The possible effects of merging more than two *pp* collisions into a single reconstructed primary vertex are assumed to be negligible in this low pile-up regime.
3. The total merging probability $p_{\text {merge}}$ for two independent reconstructible vertices is computed from the product of the merging PDF and the expected $f_{\text {exp}}(\Delta z)$ distribution:
  8 $$\begin{aligned} p_{\text {m}erge} = \int f_{\text {e}xp}(\Delta z) p_{\text {m}erge}(\Delta z) \text {d}(\Delta z). \end{aligned}$$
  It is assumed that the merging PDF for a pair of adjacent vertices $p_{\text {m}erge}(\Delta z)$ is independent of the beam conditions. The overall probability of merging two random reconstructible vertices depends on the particular beam-spot distribution, and therefore on $f_{\text {e}xp}(\Delta z)$.
4. The total number of vertices lost due to merging effects is given by:
  9 $$\begin{aligned} F(\epsilon \mu , p_{\text {m}erge}) = \epsilon \mu - \sum \limits _{\mathcal {N}_{\text {V}ertices}} P(\mathcal {N}_{\text {V}ertices}, \epsilon \mu ) \wp _{\text {m}erge}(\mathcal {N}_{\text {V}ertices}, p_{\text {m}erge}), \end{aligned}$$
  where $P(\mathcal {N}_{\text {V}ertices}, \epsilon \mu )$ is a PDF, representing the probability of reconstructing $\mathcal {N}_{\text {V}ertices}$ vertices given $\epsilon \mu$ potentially reconstructible vertices. Since the number of visible *pp* collisions varies according to Poisson with the mean of $\mu$, this function $P(\mathcal {N}_{\text {V}ertices}, \epsilon \mu )$ is a Poisson with a mean $\epsilon \mu$. The function $\wp _{\text {m}erge}(\mathcal {N}_{\text {V}ertices}, p_{\text {m}erge})$ represents the number of reconstructed vertices after taking into account merging effects, for a number, $\mathcal {N}_{\text {V}ertices}$, of vertices which would be reconstructed in the absence of any merging. This number is defined as follows:
  10 $$\begin{aligned} \wp _{\text {m}erge}(\mathcal {N}_{\text {V}ertices}, p_{\text {m}erge}) = \sum \limits _{i=1}^{\mathcal {N}_{\text {V}ertices}} p_{i}, \end{aligned}$$
  where $p_i = p_{i-1} (1-p_{i-1}p_{\text {m}erge})$, $i \ge 2$ and $p_1 = 1$. The $p_i$ represents the probability to reconstruct *i* vertices in the presence of merging effects.

### Comparison of data to simulation

To quantitatively compare data with simulation, additional effects and systematic uncertainties need to be taken into account. To account for the difference in visible cross section between data and simulation discussed in Sect. 3, the parameter $\epsilon$, extracted from the simulation fit, is scaled by a factor 1/1.11, which is equivalent to a scaling of $\mu$. A 6% uncertainty is assigned to this procedure, where the dominant contribution comes from the uncertainty in the measured value of $\mu$.

The impact of possible discrepancies in longitudinal beam-spot size between data and MC simulation was also assessed since the observed data values represent an average over a range of different and non-uniform experimental values. The MC simulation samples used in this study were generated with a beam-spot size equal to the average observed in data. The effect of a change in beam-spot size on the merging probability can be evaluated with Eq. (8). A small additional uncertainty is assigned to account for the variations of up to $\pm 2$ mm in beam-spot size in data.

**Fig. 17:**
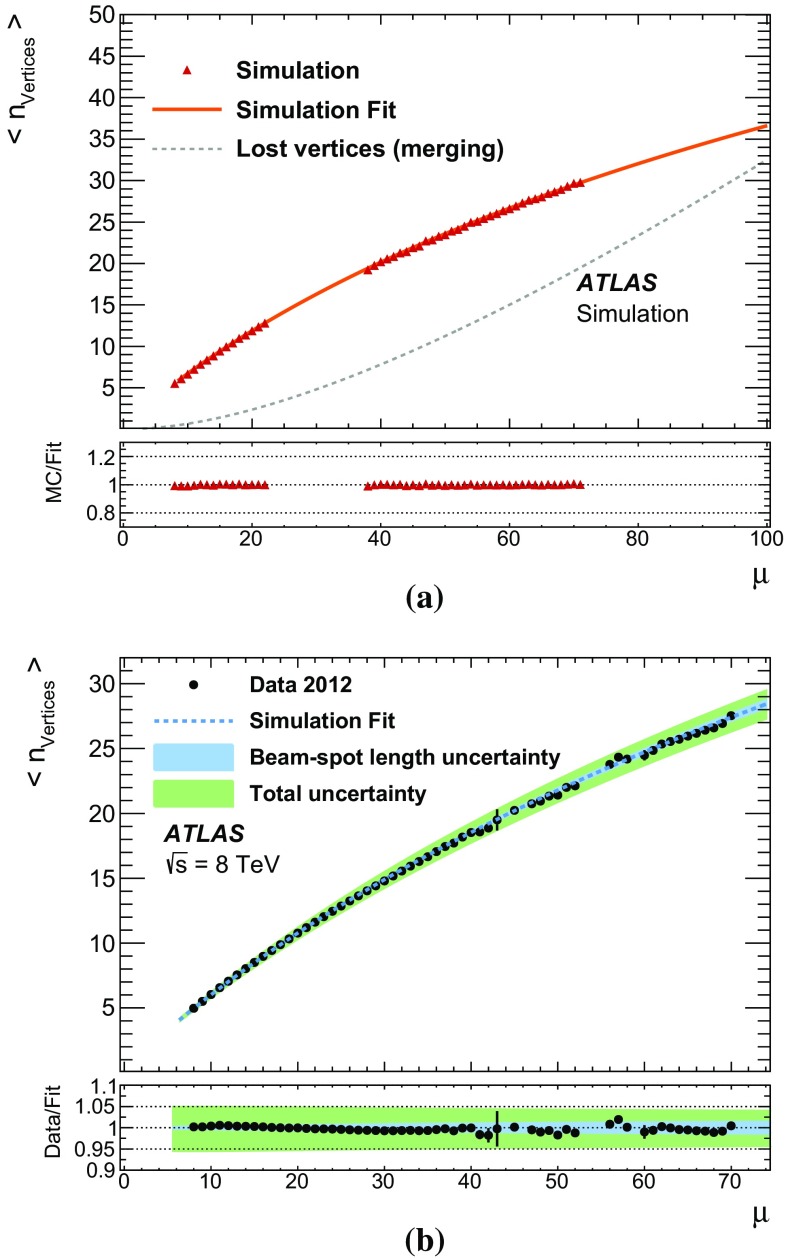
Distribution of the average number of reconstructed vertices as a function of the number of interactions per bunch crossing, $\mu$. **a** MC simulation of minimum-bias events (*triangles*) and the analytical function in Eq. (6) fit to the simulation (*solid line*). The *dashed curve* shows the average estimated number of vertices lost to merging. **b** Minimum-bias data (*black points*). The *curve* represents the result of the fit to the simulation in **a** after applying the $\mu$-rescaling correction described in the text. The *inner dark* (*blue*) band shows the systematic uncertainty in the fit from the beam-spot length, while the *outer light* (*green*) band shows the total uncertainty in the fit. The *panels* at the *bottom* of each figure represent the respective ratios of simulation **a** or data **b** to the fits described in the text

A fit using Eq. (6) was performed on MC simulation, allowing parameters $p_0$, $\epsilon$, and $p_{\text {m}erge}$ to vary. The efficiency, $\epsilon$, and merging probability, $p_{\text {m}erge}$, are extracted from the fit to simulation and found to be, $0.618 \pm 0.004{\text {(}stat.)}\pm 0.037 {\text {(}syst.)}$ and $0.0323 \pm 0.0002{\text {(}stat.)} \pm 0.0013{\text {(}syst.)}$ respectively, after correcting $\epsilon$ with the $\mu$-rescaling factor and taking into account the systematic uncertainties, as described above. The fit to MC simulation is shown in Fig. 17a.

Data are compared to Eq. (6) with the parameters $\epsilon$ and $p_{\text {m}erge}$ fixed to the values from the fit to simulation, and with the small value of $p_0$ extracted from a fit to the data. The $p_0$ parameter is irrelevant in MC simulation, which does not account for the small non-collision background present in data at low values of $\mu$. The result is shown in Fig. 17b. The uncertainty bands in Fig. 17b show the beam-spot size uncertainty and the total uncertainty, which is computed by summing in quadrature the beam-spot size and the dominant $\mu$-rescaling uncertainty terms.

The overall agreement between the data and the prediction is within 3%, with the largest observed discrepancies well within the systematic uncertainty bands.

This comparison shows that the simulation describes the primary vertex reconstruction efficiency dependence on $\mu$ accurately. Vertex merging is the effect that has the largest impact on primary vertex reconstruction efficiency as $\mu$ increases. The analytical description proposed to describe this effect is validated by the measurements based on minimum-bias data. This confirms that the main factors related to the vertex reconstruction in pile-up conditions are correctly taken into account and that the remaining effects related to the presence of fake and split vertices are negligible, as expected.

The predicted average number of reconstructed vertices, as obtained from data for a given value of $\mu$ in Fig. 17b, can be used to estimate the primary vertex selection efficiency for a specific hard-scatter process. This is done by combining the prediction with the simulated distributions of track $\sqrt{\sum p_{\text {T}} ^2}$ for this process and for minimum-bias events, as shown in Fig. 10. For the highest $\mu$ value ($\mu = 40$) studied in terms of hard-scatter primary vertex reconstruction and selection efficiencies in Sect. 5, Fig. 17b predicts an average number of reconstructed vertices from pile-up interactions of $17 \pm 1$. Of all the reconstructed vertices, the one with highest $\sum p_{\text {T}} ^2$ is selected as the hard-scatter vertex with a very high efficiency for most processes. To estimate the small probability that a pile-up vertex is selected by this procedure instead, the simulated distribution of track $\sqrt{\sum p_{\text {T}} ^2}$ for inelastic interactions in Fig. 10 is compared to the much harder one expected for the hard-scatter process of interest. For $Z \rightarrow \mu \mu$ events, a randomly selected point on the $\sum p_{\text {T}} ^2$ distribution is found to be lower than the largest of the values found for 17 random samplings of the distribution for minimum-bias events in approximately 4% of the cases. This estimate, which is partially based on data but does not account for all experimental effects such as the distortion of the track $\sum p_{\text {T}} ^2$ distribution of minimum-bias events due to merging of primary vertices, is in reasonable agreement with the estimate of 2% obtained based on simulation in Fig. 11.

## Conclusion

This paper presents primary vertex reconstruction and selection methods and their performance for proton–proton collision data recorded by the ATLAS experiment at the LHC during Run 1. The primary vertex position resolution measured in data is consistent with the predictions from simulation. A longitudinal vertex position resolution of about $30\;\upmu {\text {m}}$ has been achieved for events with high track-multiplicity. A significant improvement of the vertex transverse-position resolution is obtained using the beam-spot constraint in the vertex fit, giving a resolution below $20\;\upmu {\text {m}}$ for all multiplicities.

The primary vertex reconstruction efficiency has been measured using MC simulation. For minimum-bias events, the single vertex reconstruction efficiency is above 99% for all processes, provided at least two charged particles are reconstructed within the ATLAS inner detector. For hard-scatter interactions, the reconstruction and selection efficiency has been studied for a number of benchmark processes as a function of pile-up. In all cases, the overall signal vertex reconstruction efficiency exceeds 99%. A significant contamination from pile-up minimum-bias vertices is however observed for high values of $\mu$ in the case of hard-scatter processes with a small number of charged-particle tracks, such as $H\rightarrow \gamma \gamma$ and $Z\rightarrow \mu \mu$. The efficiency to reconstruct and then correctly select the primary vertex at $\mu = 40$ in the case of $Z\rightarrow \mu \mu$ is predicted to remain very high, namely 98%, when both muons are reconstructed within the inner detector acceptance.

The impact of multiple *pp* interactions in the same bunch crossing on the reconstruction of primary vertices has been studied in detail. Comparisons of the modelling of vertex input quantities were made for low and high values of $\mu$ and good agreement between data and the MC simulation is observed for values of $\mu$ up to 70. The largest impact of pile-up is the merging of nearby vertices, which has been quantified precisely by studying the relationship between $\mu$ and the number of reconstructed vertices. The corresponding non-linear effects due to merging are well modelled within the uncertainties in the MC simulation for values of $\mu$ as high as 70, confirming the validity of the proposed model.

## Footnotes

1 The ATLAS experiment uses a right-handed coordinate system with its origin at the nominal interaction point (IP) in the centre of the detector and the *z*-axis along the beam direction. The *x*-axis points from the IP to the centre of the LHC ring, and the *y*-axis points upward. Cylindrical coordinates $(r,\phi )$ are used in the transverse (*x*, *y*) plane, $\phi$ being the azimuthal angle around the *z*-axis. The pseudorapidity is defined in terms of the polar angle $\theta$ as $\eta =-\ln \tan (\theta /2)$.

2 Measurements of charged particle trajectories in the pixel, SCT and TRT are called *ID hits.*
